# Tobacco use and risk of COVID-19 infection in the Finnish general population

**DOI:** 10.1038/s41598-022-24148-y

**Published:** 2022-11-25

**Authors:** Sebastián Peña, Katja Ilmarinen, Laura Kestilä, Suvi Parikka, Sanna Kärkkäinen, Ida Henriette Caspersen, Ahmed Nabil Shaaban, Per Magnus, Maria Rosaria Galanti, Sakari Karvonen

**Affiliations:** 1 Department of Public Health and Welfare, Finnish Institute for Health and Welfare, Mannerheimintie 166, 00271 Helsinki, Finland; 2 Division of Insurance Medicine, Department of Clinical Neuroscience, Karolinska Institutet, Solna, Sweden; 3 Centre for Fertility and Health, Norwegian Institute of Public Health, Oslo, Norway; 4 Department of Global Public Health, Karolinska Institutet, Solna, Sweden; 5 Centre for Epidemiology and Community Medicine, Stockholm Region, Stockholm, Sweden

**Keywords:** Epidemiology, Public health

## Abstract

Empirical evidence, primarily based on hospital-based or voluntary samples, suggests that current smokers have a lower risk of COVID-19 infection than never smokers. In this study, we used nationally representative data to examine the association between tobacco use and the risk of having a confirmed COVID-19 case. We explored several forms of tobacco use, contributing to separate the role of nicotine from smoking. We used data from 44,199 participants from three pooled national health surveys in Finland (FinSote 2018–2020). The primary outcome was a confirmed COVID-19 case. We examined current smoking, moist smokeless tobacco (snus), e-cigarettes with and without nicotine and nicotine replacement therapy products. Current daily smokers had a relative risk of 1.12 of a confirmed COVID-19 case (95% CI 0.65; 1.94) in fully adjusted models compared with never smokers. Current snus use was associated with a 68% higher risk of a confirmed COVID-19 case (RR 1.68, 95% CI 1.02; 2.75) than never users. We did not find conclusive evidence of associations between e-cigarettes with and without nicotine and nicotine replacement therapy products and the risk of confirmed COVID-19 cases. Our findings suggest that nicotine might not have a protective role in the risk of COVID-19 as previously hypothesized.

## Introduction

The COVID-19 pandemic continues to spread rapidly worldwide. By October 3, 2022, there were more than 618 million confirmed SARS-COV-2 cases and more than 6.5 million deaths attributable to COVID-19^1^. Researchers have explored potential risk factors to identify patients at high risk of infection or death, as well as for targeting pharmaceutical and preventive interventions^2^.

Tobacco use, as a leading risk factor of death and disability due to respiratory diseases, was expected to increase the risk of SARS-CoV-2 infection and COVID-19 disease progression and deaths^3,4^. Smokers have generally increased risk of other respiratory infections and could be expected to have higher risk of SARS-CoV-2 due to repetitive hand-to-mouth handlings, increased mask handlings^5^, sharing of cigarettes and vape devices^6^ and creation of aerosols which might be carriers of viruses. On the other hand, smokers might have fewer social contacts^7^ and be less exposed to indoor places^8^. Studies have also reported mixed findings on whether tobacco or nicotine could modify the expression of ACE2 receptors, which provide a cellular entry point for SARS-CoV-2^9^.

Earlier epidemiological studies showed that smokers were underrepresented among patients hospitalized due to COVID-19^10^. These results could be explained by the selected nature of the sample or information bias arising from data collected retrospectively or from electronic health records^11,12^. However, the most recent meta-analysis, including more diverse samples and study designs, has confirmed these early findings showing that current smokers had lower risk of COVID-19 than never smokers (Relative risk 0.67, 95% Credible interval 0.60; 0.75)^13^.

A message of a protective effect of tobacco use could undermine public health efforts to curb its use and reduce the perception of harm in the general population^14^. Studies with general population samples, with a lower risk of selection bias, are thus urgently needed.

Finland is a pioneer in tobacco control. Since the 1970s, Finland has consistently introduced legislation to increase prices and restrict tobacco availability and marketing^15^. By 2018, 14% of Finns aged 15 or older were daily smokers and 2.2% were regular e-cigarettes users, both indicators below OECD average^16^. The prevalence of daily or occasional use of snus was 3% in 2018^17^.

The aim of the study was to examine the association between tobacco use and the risk of having a confirmed COVID-19 case. We explored several forms of tobacco use (smoking, snus, e-cigarettes with and without nicotine and nicotine replacement therapy products) and investigated whether introducing a potential collider bias by adjusting for a mediating risk factor (i.e. body mass index, BMI) could induce a spurious association. We used data from nationally representative health surveys in Finland linked to data on confirmed COVID-19 cases, which is less subject to selection bias than voluntary-based samples^18,19^.

## Methods

We registered the study in ClinicalTrials.gov (NCT04915781). Changes to protocol are described in detail in the Supplementary Appendix. In brief, the main change was that we analysed the data as cross-sectional (and not as a prospective cohort study) because data from FinSote 2020 was collected after the start of the pandemic. We were not able to adjust for physical activity as a potential collider and decided to exclude alcohol use as a potential collider due to its less clear causal relationship with tobacco use. We merged daily and occasional users of other forms of tobacco and nicotine than smoking to increase the statistical power. We report the study in accordance with the Strengthening the Reporting of Observational Studies in Epidemiology (STROBE) statement^20^.

### Setting and study design

The design is an observational study of pooled cross-sectional population health surveys in Finland linked to data on a confirmed case of COVID-19 (i.e. a positive PCR testing of SARS-CoV-2, either reported by a laboratory or a physician). The study populations were permanent residents in Finland from the FinSote surveys (2018, 2019 and 2020). We linked survey data to confirmed COVID-19 cases from the Communicable Diseases Registry until August 23, 2021, using a unique personal identifier assigned to all Finnish residents.

### Data sources

We pooled data from three cross-sectional population health surveys in Finland. The FinSote 2017–2018 Survey was a nationally representative survey of the Finnish population aged 20 years and over. The sampling frame was the Population Register of Statistics Finland^21^. The survey was based on a stratified random sampling. In 2017, 3300 people were invited to participate from each of 18 research areas (2300 adults aged 20–74 and 1000 adults aged 75 +, total sample size 59,400). Data was collected between October 2, 2017, and March 3, 2018. Participants received a self-administered questionnaire in Finnish, Swedish, English and Russian, which could be returned on paper or electronically. The participation rate was 45%. We excluded participants who did not provide consent for register linkage, resulting in an analytical sample of 14,736 participants^21^.

FinSote 2019 was a nationally representative survey of the Finnish population aged 15 and over, which was implemented in conjunction with the European Health Information Survey (EHIS) round 3. The sampling frame was the Population Register of Statistics Finland. The survey was based on a random sample of 15,000 individuals. Participants received a self-administered questionnaire available in Finnish, Swedish and English, which could be returned in paper or electronically. All participants consented to record linkage. The participation rate was 44%, resulting in an analytical sample of 6251 participants^22^.

FinSote 2020 was a nationally representative survey of the Finnish population aged 20 and over. The sampling frame was the Population Information System from the Digital and Population Data Services Agency, created in January 2020 after the merge between the Population Register of Statistics Finland and local register offices^23^. The survey was based on a stratified random sample from each of 22 regions (2000 adults aged 20–74, 800 adults aged 75 +, total sample size 61,600). Data collection started on September 14, 2020, and finished on February 8, 2021. Participants received a self-administered questionnaire in Finnish, Swedish, English and Russian, which could be returned on paper or electronically. All participants consented to record linkage. The analytical sample was 28,199 participants, with a participation rate of approximately 46%.

All surveys comply with the Declaration of Helsinki regarding confidentiality, anonymity, and data protection. FinSote surveys were approved by the institutional review board of the Finnish Institute for Health and Welfare (decision THL/637/6.02.01/2017). Informed consent was obtained from all participants.

### Outcomes

The primary outcome was a confirmed COVID-19 case, defined here as those cases with a positive SARS-CoV-2 RT-PCR, either informed by a laboratory or by a physician as a record of an ICD-10 code U07.1 (which requires a positive SARS-CoV-2 RT-PCR), following national guidelines^24^. Reporting of COVID-19 positive cases was mandatory for laboratories and physicians throughout the study period^25^. We obtained COVID-19 infection data until August 23, 2021, from the Finnish National Infectious Disease Register maintained by the Finnish Institute for Health and Welfare. Testing of SARS-CoV-2 is free in Finland. Coverage has been extensive and the total number of tests exceeds 6.4 million by August 23, 2021^26^.

### Exposure variables

The exposure of interest was tobacco use. All FinSote surveys had comparable questions on smoking. Comparable questions on snus use were available for people aged 20–74 in FinSote 2018 and 2020 and all participants in FinSote 2019. FinSote 2018 and 2020 included questions for people aged 20–74 about electronic cigarettes with and without nicotine and nicotine replacement therapy products. For smoking, we created a categorical variable with the following categories: never smokers, former smokers, current occasional smokers and current daily smokers. We categorised snus use, e-cigarettes with or without nicotine and nicotine replacement therapy products in a similar way: never users, former users and current users. More details on the specific questions and harmonization strategy can be found in the Supplementary Appendix.

### Confounders

**Figure 1:**
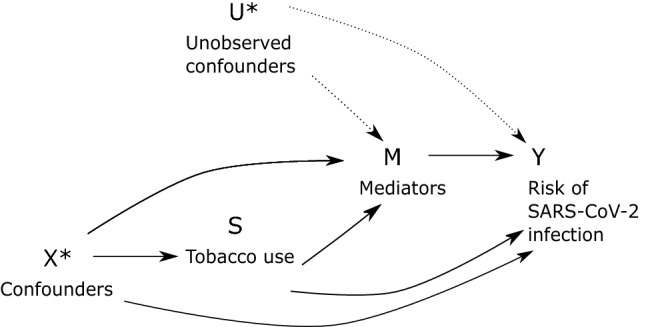
Directed acyclic diagram of the study. *Note*: Confounders X* in this study are sex, age, marital status, years of education, mother tongue and participation in social activities. Unobserved mediator-exposure confounders U* include, for example, genetic factors associated with an increased risk of obesity and SARS-CoV-2 infection but not tobacco use. Mediators and potential colliders M include BMI (observed), and other unobserved factors, such as SARS-CoV-2 testing or chronic health conditions caused by tobacco use.

We adjusted for covariates that we considered a priori that causally precede the exposure and are associated with the outcome^27^. Evidence shows clear associations with sociodemographic factors and COVID-19 incidence in Finland and other settings^28–30^. Tobacco use is also associated with demographic and social factors, including social capital and social participation^31^. As a result, we adjusted for sex, age, marital status, years of education, mother tongue, and participation in social activities drawing on the directed acyclic graph shown in Fig. 1. Data from FinSote 2018 and 2019 was collected prior to the COVID-19 pandemic, while data from FinSote 2020 was collected during the pandemic.

We used age as a continuous variable. We defined marital status as those married, in a registered relationship or cohabiting versus those separated or divorced, widowed or single. We measured years of education as the number of years a person has attended school or studied full time altogether. We obtained information on the participant's mother tongue from national registries and categorized it into Finnish, Swedish and others. We measured participation with a question about participation in the activities of any club, association, hobby group or religious or spiritual community. We categorised participation into the following: no participation, occasional and active.

In addition, given participants living in different regions in Finland have varying risks of COVID-19 due to geographical variation in viral spread and diverging public health and social distancing measures, we included fixed effects for hospital districts (20) to account for these variations. We chose these administrative units because this is the level used to define COVID-19 epidemic phases.

### Collider bias

We examined whether inducing potential collider bias (M-bias) by adjusting for other behavioural risk factors could explain earlier results^11,12^. We tested the effect of collider bias using BMI as an exemplary case. In the case of BMI, several unmeasured factors could induce mediator-outcome confounding and, thus, create collider bias. This includes, for example, genetic factors associated with BMI and risk of COVID-19, but not related to tobacco use. Other potential colliders, such as chronic conditions caused by tobacco use (e.g., chronic bronchitis) and SARS-CoV-2 testing or hospitalizations were not included due to lack of data.

We calculated body mass index as the self-reported weight (in kg) divided by height (in m) squared and created a continuous variable. We considered as extreme outliers participants who reported values of height outside the range of FINRISK 2012, which is a national health examination survey with standardized measurement techniques (range values 137–218). We excluded those extreme outliers from the analyses.

### Risk of bias

The study was observational and we did not have a source of exogenous variation to obtain causal estimates. We used the conditional independence assumption to approximate causal estimates^32,33^, as well as correctly defining confounders to prevent collider bias. The identifying assumption of conditional independence states that after conditioning on a set of observable covariates (i.e., confounders $X^{*}$), exposure to tobacco was independent of potential outcomes. In other words, after controlling for confounders, exposure to tobacco was assumed to be randomly assigned. This is a strong assumption, as there might be unobserved factors $U$ leading to residual confounding (e.g., certain personality traits, such as lower risk aversion, could increase the risk of tobacco use and increase the risk of COVID-19 due to lower adherence to social distancing restrictions, as well as other unobserved factors as religion or certain hobbies (e.g. singing in a choir), that might confound the association between tobacco use and COVID-19 infection.

### Statistical analyses

We used Poisson regression with robust standard errors to estimate the relative risk of a confirmed COVID-19 case^34^. The use of data from the year 2020 (collected after the start of the COVID-19 pandemic) precluded the use of hazard ratios as preregistered. We report analyses of FinSote 2018 and 2019 using time-to-event data in sensitivity analyses as registered in the protocol (see Supplementary Appendix).

We fitted the following Poisson model:

1 $$log \mu_{i} = \beta_{0} + \beta_{1} S_{i} + \beta_{x} X_{i}^{*} + \rho_{1} R_{a}$$

where $i$ denotes the individual, $\beta_{0}$ is the intercept; $\beta_{1}$ is the coefficient of interest for exposure to tobacco $S$; a vector of covariates $X^{*}$ (i.e., sex, age, marital status, years of education, mother tongue and participation in social activities); and fixed effects $\rho_{1} R_{a}$ for region $a$.

We tested for non-linearity in the association between continuous variables (age, years of education, and BMI) and the outcome by comparing the linear model with penalized smoothing splines^35^ using a likelihood ratio test with the Wald method^36^. The likelihood ratio test showed a better fit using penalized smoothing splines for all three continuous variables. We therefore modelled them using penalized smoothing splines. We tested the possibility of collider bias due to behavioural risk factors by assessing the change in the coefficient $\beta_{1}$ after adjusting for BMI in the model fully adjusted for confounders.

We carried out three sensitivity analyses: (i) we conducted separate analyses including only FinSote 2018 and 2019, since this data was collected prior to the COVID-19 pandemic and is thus less subject to information bias; (ii) we re-conducted the analyses in (i) but using Cox proportional hazard models to adhere to the registration protocol; and (iii) we re-conducted the main analyses but excluding users of other forms of tobacco (e.g. in analyses of smoking we excluded current users of snus, e-cigarettes with nicotine and nicotine replacement therapy products). More details are provided in the Supplementary Appendix. We carried out an additional post-hoc stratified analyses by vaccination period to explore whether the association between tobacco use and COVID-19 incidence changed after the start of the rollout of COVID-19 vaccinations on December 26, 2020. We restricted these analyses to smoking and snus use due to the small number of cases for the other forms of tobacco use.

We used R version 3.6.3 for all analyses. We used the *svyglm* functions in the *survey* package to fit the Poisson regression considering the complex sampling design in all analyses. An annotated statistical code can be found in the Supplementary Appendix.

## Results

A total of 44,199 participants with recorded smoking status were included in the study (after excluding 4987 participants, 10.1%, with incomplete data). Table S1 shows the proportions of missing data for each variable and survey year. Current daily smokers were more often male, younger, had lower years of education and reported lower active participation in social activities than never smokers (Table 1). Tables S2-S5 shows the baseline characteristics by other forms of tobacco use.

**Table 1:** Baseline characteristics of 44,199 participants of FinSote 2018, 2019 and 2020 by smoking status.

	Never smoker	Former smoker	Occasional smoker	Daily smoker
n	19,438	18,826	2129	3806
Confirmed COVID-19 cases, %	176 (44.6)	156 (39.5)	29 (7.3)	34 (8.6)
Sex, % female	59.3	46.9	41.3	45.6
Mean age, (SD)	49.4 (19.4)	52.5 (17.4)	40.2 (15.0)	46.7 (15.4)
Marital status, % separated, single or widowed	36.7	29	42.1	41.6
Mean years of education (SD)	14.4 (4.1)	13.7 (4.1)	14.4 (3.7)	13.0 (3.6)
**Mother tongue, %**				
Finnish	94.2	91.8	93.2	94.4
Swedish	4	6.1	5	4
Other	1.8	2	1.8	1.5
**Participation in social activities, %**				
No participation	44.5	49.8	53	67.2
Active	31	27	23.5	14.9
Occasional	24.5	23.2	23.5	17.9
Mean BMI (SD)	26.3 (5.2)	27.2 (5.2)	26.8 (5.1)	27.0 (5.5)

Data are means (standard deviation) and percentages. Means and percentages incorporate complex survey design.

In our full sample, 395 participants with complete data had a confirmed COVID-19 case. Current daily smokers had a relative risk of 1.12 of a confirmed COVID-19 case (95% CI 0.65; 1.94) in the model fully adjusted for confounders compared with never smokers (Table 2). The estimates had wide confidence intervals and were compatible with a large range of associations. The relative risk for current occasional and former smokers were 0.73 (95% 0.44; 1.22) and 1.06 (95% CI 0.78; 1.45), also compatible with a wide range of associations (Table 2).

**Table 2:** Relative risk of confirmed COVID-19 cases by tobacco use in participants of FinSote surveys.

	COVID-19 cases	Relative risk (95% CI)		
		Model 1: Adjusted for age and sex	Model 2: Model 1 and all confounders	Model 3: Model 2 and potential collider
**Smoking status (n = 44,199)**				
Current daily smoker	34	1.12 (0.64; 1.97)	1.12 (0.65; 1.94)	1.11 (0.64; 1.92)
Current occasional smoker	29	0.72 (0.44; 1.19)	0.73 (0.44; 1.22)	0.72 (0.43; 1.19)
Former smoker	156	1.06 (0.76; 1.46)	1.06 (0.78; 1.45)	1.05 (0.77; 1.44)
Never smoker	176	Ref	Ref	Ref
**Snus use (n = 32,931)**				
Current user	30	1.58 (0.96; 2.59)	1.68 (1.02; 2.75)	1.65 (1.01; 2.70)
Former user	47	1.01 (0.63; 1.63)	1.09 (0.7; 1.71)	1.08 (0.68; 1.70)
Never user	292	Ref	Ref	Ref
**E-cigarette with nicotine (n = 27,098)**				
Current user	4	2.05 (0.59; 7.15)	2.26 (0.66; 7.69)	2.18 (0.66; 7.16)
Former user	42	0.95 (0.58; 1.55)	0.94 (0.57; 1.53)	0.92 (0.56; 1.51)
Never user	231	Ref	Ref	Ref
**E-cigarette without nicotine (n = 27,005)**				
Current user	1	0.22 (0.03; 1.66)	0.24 (0.03; 2.04)	0.23 (0.03; 1.90)
Former user	43	1.27 (0.77; 2.09)	1.23 (0.74; 2.04)	1.21 (0.73; 2.0)
Never user	233	Ref	Ref	Ref
**Nicotine replacement therapy products (n = 27,073)**				
Current user	16	1.36 (0.64; 2.88)	1.45 (0.69; 3.03)	1.44 (0.69; 3.01)
Former user	47	1.02 (0.64; 1.62)	1.0 (0.62; 1.61)	1.01 (0.63; 1.63)
Never user	210	Ref	Ref	Ref

Estimates for smoking include all age groups and all FinSote surveys. Estimates on snus use include participants 20–74 years old in FinSote 2018 and 2020 and all age groups in FinSote 2019. Estimates on e-cigarettes and nicotine replacement products include all age groups in FinSote 2018 and 2020.

Model 2 adjusted for marital status, years of education, mother tongue, and participation in social activities. Model 3 adjusted additionally for BMI. Analyses incorporate complex sampling design.

Current snus use was associated with a 68% higher risk of a confirmed COVID-19 case (RR 1.68, 95% CI 1.02; 2.75) than never users (Table 2). The relative risk for former users was 1.09 (95% CI 0.70; 1.71). There were very few confirmed cases of COVID-19 among current users of e-cigarettes (with or without nicotine) and nicotine replacement therapy products, resulting in very imprecise estimates.

Adjusting for BMI as a potential collider resulted in small attenuations of the point estimates but did not substantially change the results (Table 2). For example, the point estimate of the relative risk of COVID-19 for current daily smokers changed from 1.12 in the model fully adjusted for confounders to 1.11 in the model additionally adjusted for BMI.

Sensitivity analysis restricting the data to 2018 and 2019 were not informative due to very imprecise estimates (Table S6). Sensitivity analyses using Cox proportional hazard models yielded almost identical estimates as Poisson regression models (Table S7). Excluding users of other forms of tobacco resulted in similar estimates than the main analyses (Tables S8 and S9). Exploratory post-hoc analyses by vaccination period showed that daily smokers had a lower risk of COVID-19 in the period pre-COVID-19 vaccination (fully adjusted RR 0.27, 95% CI 0.10; 0.74) and a higher risk of COVID-19 in the period after the rollout of COVID-19 vaccinations (fully adjusted RR 1.52, 95% CI 0.83; 2.79). Current snus users had a higher risk of COVID-19 during both periods, consistent with the main findings.

## Discussion

We examined the association between different forms of tobacco use and the risk of having a confirmed COVID-19 case. We did not find evidence that smoking (current daily, occasional and former smoking) is associated with the risk of a COVID-19 case. Our estimates are weakly informative of an increased risk of confirmed COVID-19 among current daily smokers and former smokers and a lower risk of confirmed COVID-19 among current occasional smokers compared to never smokers. Current snus use was associated with a higher risk of having a confirmed COVID-19 case. Results for e-cigarettes (with or without nicotine) and nicotine replacement therapy products were inconclusive. Post-hoc sensitivity analyses showed that smokers had a lower risk of COVID-19 in the period before the start of COVID-19 vaccination rollout and higher risk after the start of COVID-19 vaccinations.

We did not find evidence of an association between smoking and the risk of a confirmed COVID-19 case in the whole study period. The results of the largest living systematic review and meta-analysis to date showed that current smokers had 33% lower risk of SARS-CoV-2 infection than never smokers (RR 0.67, 95% credible intervals 0.60; 0.75)^13^. However, only seven out of 39 studies included in the meta-analysis were carried out in random or nationally representative samples and were considered of good quality^37–43^. All these studies were seroprevalence studies in national or subnational random samples and showed a negative association between current smoking and seroprevalent SARS-CoV-2. However, they reported unadjusted^42^ or minimally adjusted estimates^38,39,41,44^ and none controlled for socioeconomic status, making residual confounding highly likely. Our post-hoc findings of the pre-vaccination period are consistent with these findings. In the post-vaccination period, weak evidence of a higher risk of COVID-19 among smokers could be explained by lower vaccine adherence among smokers.

We found that current snus users had a higher risk of confirmed COVID-19. Comparison with previous studies is limited as, to our knowledge, no study has examined the association between snus use and the risk of COVID-19. Snus is a commonly used tobacco product in Nordic countries. While snus sales are banned in Finland, imports are allowed for personal use. In Finland, snus is primarily used by young men^17^, with higher use among Swedish-speaking Finns and active sports players (especially ice hockey)^45^. Other forms of nicotine exposure have been examined in few studies. A study in the United States in adolescents and young adults aged 13–24 did not find conclusive evidence of an association between e-cigarettes and COVID-19 positive diagnosis (OR 1.9, 95% CI 0.8; 4.7)^46^. Dual users (i.e. cigarettes and e-cigarettes) had 6.8 higher odds of COVID-19 diagnosis (95% CI 2.4; 19.6)^46^. Another study in the United Kingdom also did not find conclusive results on the association between current e-cigarette use and diagnosed or suspected COVID-19^47^. Our results suggest that nicotine does not play a protective role in the risk of infection from SARS-CoV-2. While we cannot rule out the existence of biological effects of snus use, our findings suggest that social and environmental mechanisms increasing viral exposure outweigh these hypothetical protective effects.

Adjusting for BMI as a potential collider did not change substantially the results. In a large population study in the United Kingdom, adjusting for BMI, smoking, index of multiple deprivation, and comorbidities resulted in a change in direction of the effect (hazard ratios changed from 1.14 to 0.89)^2^. Post-hoc analyses showed that this was mainly due to adjusting for chronic respiratory disease, which is a consequence of smoking and might have induced collider bias^2^. We were only able to adjust for BMI, and not for chronic conditions, which might explain why we did not observe a similar change.

Our results come from a setting of relatively low COVID-19 viral spread. During the first wave in 2020, Finland introduced a nationwide closure of schools, restaurants and mass gatherings, without resorting to statutory lockdown policies. In May 2020, the country transitioned to a so-called “hybrid strategy” which included broad testing-tracing-isolation actions, targeted regional measures and vaccination rollout (which started in December 26, 2020)^48^. This should be considered when assessing the external validity of our findings, which are mostly generalizable to other Nordic countries and high-income countries with low levels of tobacco use and COVID-19 viral spread. In addition, as suggested by our exploratory analyses by vaccination period, it is possible that the risk of COVID-19 among tobacco users varied over time. Further studies should explore this hypothesis in more depth.

Major strengths in our study included the use of pooled nationally representative data and the relatively large sample size. The sampling frame includes all permanent residents in Finland, including people living in institutions and conscripts, which reduces the risk of selection bias. The outcome is measured using standardized techniques and case definitions and we consider it has a lower risk of misclassification bias. Finally, we were able to control for a larger set of confounders than previous studies, reducing (although not completely) the risk of residual confounding.

However, some limitations are noted. First, there were very few COVID-19 cases in our data, resulting in a low statistical power to observe such a weak association between smoking and the risk of COVID-19. This reflects the fact that Finland has been relatively effective in controlling the viral spread during the COVID-19 pandemic. Up to August 23, 2021, Finland has had less than 125,000 cases^1^. We consider, however, that the study is worth doing even if the results are only weakly informative, as we are exploring several other forms of tobacco and nicotine use and our estimates can be meta-analysed with future studies to obtain more precise estimates^49^. In addition, COVID-19 cases are likely underestimated. This underestimation was larger during the first months of the COVID-19 pandemic, where testing was restrictive to health workers and hospitalized patients. As part of a change to a hybrid policy, testing was expanded significantly in May 2020^50^. As a result, positivity rates went from 9.7% in March 2020 to 2.0% in May 2020 and remained below 4% throughout the study period^51^. Underestimation would only bias our results if exposure groups would differ in their access or use of testing. We consider this possibility as low, as testing was accessible and free of charge, but we cannot rule this out. Second, part of our data was collected during the COVID-19 pandemic and, in some cases, after participants have had COVID-19, which might have influenced their responses to the questions on smoking and other behavioural risk factors. Our sensitivity analyses restricted to 2018 and 2019 were not informative due to very imprecise estimates. Third, given the time lag between exposure and outcome assessment, it is possible that some current smokers in 2018 or 2019 might have quit smoking at the start of the pandemic, introducing misclassification bias in the measurement of exposure. This bias, however, is likely to be small as the time lag is relatively short. Fourth, we were not able to adhere to our pre-registered analysis plan of analysing the data as time-to-event data. However, SARS-CoV-2 is a new pathogen and participants were considered at-risk at the start of the pandemic in Finland (i.e., February 27), creating a unique situation where all participants who did not experience the outcome have identical follow-up time. This leads to a discrete Cox model that in practice provides similar estimates to our current analysis^52^.

## Conclusions

We did not find conclusive evidence of an association between current smoking and the risk of a COVID-19 case. Current snus users had a higher risk of a confirmed COVID-19 case. Our findings suggest that nicotine might not have a protective role in the risk of SARS-CoV-2 infection as previously hypothesized. Future research could use instrumental variable designs, such as Mendelian randomization, to obtain more robust causal effects, and explore the role of COVID-19 vaccinations on the association between tobacco use and risk of COVID-19 and adverse outcomes.

## Supplementary Information

Supplementary Information 1.

Supplementary Information 2.

## Data Availability

The data used in this study are not publicly available but can be accessed with a research proposal and an approved user authorisation application from the Finnish Social and Health Data Permit Authority (Findata). For more information, please visit https://findata.fi/en/.
