# Supplementary material for: Tobacco use and risk of COVID-19 infection in the Finnish general population
Source: Sci Rep. 2022 Nov 25;12:20335. doi: 10.1038/s41598-022-24148-y (PMC9700668; doi:10.1038/s41598-022-24148-y)
Supplement: Supplementary file 1 — Supplementary Information 1. [file 41598_2022_24148_MOESM1_ESM.docx]

**Supplementary Appendix for the article:**

**Tobacco use and risk of COVID-19 infection in the Finnish general population**

Sebastián Peña, Katja Ilmarinen, Laura Kestilä, Suvi Parikka, Sanna Kärkkäinen, Ida Henriette Caspersen, Ahmed Nabil Shaaban, Rosaria Galanti, Per Magnus, Sakari Karvonen

**Correspondence:**

Sebastián Peña

Finnish Institute for Health and Welfare

Mannerheimintie 166

Helsinki

FINLAND

Phone: +358 45 245 1360

Email: sebastian.penafajuri@thl.fi

[**Changes in the design, definitions and methods compared to the registered protocol**](#_8lb1g57h6exz) **3**

[**Harmonisation of FinSote survey questions**](#_mqutv4yv08ek) **4**

[2.1 Exposures](#_r041g18m5lms) 4

[2.2 Confounders](#_670xmtem71yz) 9

[2.3 Potential collider](#_pcrts65ux3l) 11

[**Missing data in FinSote 2018-2020**](#_x8c5zz2i9gdj) **13**

[Table S1. Proportion of missing data by variables and survey year](#_ofswkpxvbvt7) 13

[**Baseline characteristics of FinSote participants by other forms of tobacco use**](#_fo5wi8e9kklh) **14**

[Table S2. Baseline characteristics of 32931 FinSote participants by snus use](#_mzaippx674kz) 14

[Table S3. Baseline characteristics of 27098 FinSote participants by e-cigarette with nicotine use](#_p9lj69j6xno) 15

[Table S4. Baseline characteristics of 27005 FinSote participants by e-cigarette without nicotine use](#_nbtdnjtfm37l) 16

[Table S5. Baseline characteristics of 27073 FinSote participants by nicotine replacement product use](#_hsmr7jfz3gyu) 17

[**Sensitivity analyses using data from FinSote 2018 and 2019**](#_aduf84nnlse6) **18**

[Table S6. Relative risk of confirmed COVID-19 cases by tobacco use in participants of FinSote surveys 2018 and 2019](#_ak93lw87twnu) 18

[Table S7. Hazard ratios of confirmed COVID-19 cases by tobacco use in participants of FinSote surveys 2018 and 2019](#_4i5i3urkgszt) 21

[Table S8. Relative risk of confirmed COVID-19 cases by tobacco use in participants of FinSote surveys after excluding other tobacco users](#_a9vlt42df1a) 23

[Table S9. Relative risk of confirmed COVID-19 cases by tobacco use in participants of FinSote surveys after excluding other tobacco users and those with missing data on tobacco products](#_dlkovnf4gbjm) 24

[Table S10. Relative risk of confirmed COVID-19 cases by tobacco use in participants of FinSote surveys by time periods (before and after the start of COVID-19 vaccination rollout)](#_jekd0qd3vkeq) 25

# Changes in the design, definitions and methods compared to the registered protocol

- We registered the study in ClinicalTrials.gov (NCT04915781) on June 7, 2021. We only started data wrangling and exploratory data analysis after registration, and as a result we introduced the following changes:
- **Design**. We realised that the inclusion of FinSote 2020 (which data was collected during the COVID-19 pandemic) did not allow us to examine the incidence of a confirmed COVID-19 case. Instead of a cohort study, the study design is an observational study with a mix of data collected prospectively (prospective cohort study) and data collected retrospectively/concurrently (cross-sectional study). We included in sensitivity analyses the analyses of FinSote 2018-2019 which adhere to the pre-established protocol.
- **Exposures**. During the exploratory data analysis, we noticed that the frequency of users of other forms of tobacco was low as well as the number of COVID-19 cases among these users. We decided, thus, to combine daily and occasional users into a single category of “current users” to increase the statistical power.
- **Collider bias analysis**. Questions for physical activity were different in the year 2020. In principle, these could be combined into a harmonised variable, but during exploratory data analysis, it was clear that the distribution of the responses was very different. We, therefore, excluded physical activity from the analyses. We also decided to use only body mass index as a potential collider as there were clearer potentially unobserved mediator-outcome confounders that could create collider bias, whereas the causal structure for alcohol use was less clear.

# Harmonisation of FinSote survey questions

We harmonised the three FinSote surveys using a structured protocol. The original forms in Finnish, Swedish, English and Russian are available [here](https://thl.fi/fi/tutkimus-ja-kehittaminen/tutkimukset-ja-hankkeet/finsote-tutkimus/miten-finsote-tutkimus-tehdaan/lomakkeet).

## ***2.1 Exposures***

2.1.1 Smoking status

Variable: smoking_status

Type: Factor, [“never smoker”, “former smoker”, “current occasional smoker”, “current daily smoker”]

Description. Smoking was asked in FinSote 2018 and 2020 with the question “Do you smoke currently (cigarettes, cigars or pipe)?”. FinSote 2019 was implemented together with EHIS 3 and thus questions were different but still very comparable. The first question asked “Do you smoke at present (other tobacco products than e-cigarettes)?” which allowed us to separate daily and occasional smokers. If the answer was “not at all”, respondents would be directed to another question “Have you ever smoked daily for a period of at least one year? For how many years altogether?” which allows us to separate former and never smokers.

Questions, answers and equivalence are shown below

| **Survey** | **Question** | **Answer** | **Equivalence** |
| --- | --- | --- | --- |
| FinSote 2018 | 84. Do you smoke currently (cigarettes, cigars or pipe)? | 1.yes, daily  2. occasionally  3. not at all  4. I have never smoked | Current daily smoker 1  Current occasional smoker 2  Former smoker 3  Never smoker 4 |
| FinSote 2019 | 73. Do you smoke at present (other tobacco products than e-cigarettes)?  76. Have you ever smoked daily for a period of at least one year? For how many years altogether? | 1. yes, daily  2. Yes, occasionally  3. not at all  1. I have never smoked daily  2. I have smoked daily for a total of _ years | Current daily smoker Q73=1  Current occasional smoker Q73=2  Former smoker Q73=3 & Q76=2  Never smoker Q73=3 & Q76=1 |
| FinSote 2020 | 62. Do you smoke currently (cigarettes, cigars or pipe)? | 1.yes, daily  2. occasionally  3. not at all  4. I have never smoked | Current daily smoker 1  Current occasional smoker 2  Former smoker 3  Never smoker 4 |

2.1.2 Snus status

Variable: snus_status

Type: Factor, [“never user”, “former user”, “current user”]

Description. Snus use was asked in FinSote 2018 and 2020 with a question “Do you currently use any of the following products? snus (Swedish type moist snuff)” and in FinSote 2019 as “Do you currently use snuff?”. This question was not available in FinSote 2018 and 2020 in the questionnaire for 75+ as it was assumed snus use in this age category is very uncommon.

Questions, answers and equivalence are shown below:

| **Survey** | **Question** | **Answer** | **Equivalence** |
| --- | --- | --- | --- |
| FinSote 2018 | 85a. Do you currently use any of the following products?  snus (Swedish type moist snuff) | 1. yes, daily  2. occasionally  3. not at all  4. I have never used | Current user 1 or 2  Former user 3  Never user 4 |
| FinSote 2019 | 78. Do you currently use snuff?  76. Have you ever smoked daily for a period of at least one year? For how many years altogether? | 1. yes, daily  2. yes, occasionally  3. not at all  4. I have never used it | Current user 1 or 2  Former user 3  Never user 4 |
| FinSote 2020 | 63a. Do you currently use any of the following products?  snus (Swedish type moist snuff) | 1. yes, daily  2. occasionally  3. not at all  4. I have never used | Current user 1 or 2  Former user 3  Never user 4 |

2.1.3 E-cigarettes with nicotine

Variable: ecig_nic_status

Type: Factor, [“never user”, “former user”, “current user”]

Description. The use of e-cigarettes with nicotine was asked in FinSote 2018 and 2020 with a question “Do you currently use any of the following products? e-cigarettes with nicotine”. FinSote includes a question on general use of e-cigarettes “Do you currently use electronic cigarettes (e-cigarettes)?” but we considered it necessary to separate whether they contained nicotine as it is a crucial component of the research question. This question was not available in FinSote 2018 and 2020 in the questionnaire for 75+ as it was assumed e-cigarette use in this age category was very uncommon.

Questions, answers and equivalence are shown below:

| **Survey** | **Question** | **Answer** | **Equivalence** |
| --- | --- | --- | --- |
| FinSote 2018 | 85b. Do you currently use any of the following products?  e-cigarettes with nicotine | 1. yes, daily  2. occasionally  3. not at all  4. I have never used | Current user 1 or 2  Former user 3  Never user 4 |
| FinSote 2020 | 63b. Do you currently use any of the following products?  e-cigarettes with nicotine | 1. yes, daily  2. occasionally  3. not at all  4. I have never used | Current user 1 or 2  Former user 3  Never user 4 |

2.1.4 E-cigarettes without nicotine

Variable: ecig_nonic_status

Type: Factor, [“never user”, “former user”, “current user”]

Description. The use of e-cigarettes without nicotine was asked in FinSote 2018 and 2020 with a question “Do you currently use any of the following products? e-cigarettes without nicotine”. This question was not available in FinSote 2018 and 2020 in the questionnaire for 75+ as it was assumed e-cigarette use in this age category was very uncommon.

Questions, answers and equivalence are shown below:

| **Survey** | **Question** | **Answer** | **Equivalence** |
| --- | --- | --- | --- |
| FinSote 2018 | 85c. Do you currently use any of the following products?  e-cigarettes without nicotine | 1. yes, daily  2. occasionally  3. not at all  4. I have never used | Current user 1 or 2  Former user 3  Never user 4 |
| FinSote 2020 | 63c. Do you currently use any of the following products?  e-cigarettes without nicotine | 1. yes, daily  2. occasionally  3. not at all  4. I have never used | Current user 1 or 2  Former user 3  Never user 4 |

2.1.5 Nicotine replacement therapy products

Variable: nrt_status

Type: Factor, [“never user”, “former user”, “current user”]

Description. The use of nicotine replacement products was asked in FinSote 2018 and 2020 with a question “Do you currently use any of the following products? nicotine replacement therapy products such as patches or chewing gum”. This question was not available in FinSote 2018 and 2020 in the questionnaire for 75+.

Questions, answers and equivalence are shown below:

| **Survey** | **Question** | **Answer** | **Equivalence** |
| --- | --- | --- | --- |
| FinSote 2018 | 85d. Do you currently use any of the following products?  nicotine replacement therapy products such as patches or chewing gum | 1. yes, daily  2. occasionally  3. not at all  4. I have never used | Current user 1 or 2  Former user 3  Never user 4 |
| FinSote 2020 | 63d. Do you currently use any of the following products?  nicotine replacement therapy products such as patches or chewing gum | 1. yes, daily  2. occasionally  3. not at all  4. I have never used | Current user 1 or 2  Former user 3  Never user 4 |

## **2.2 Confounders**

2.2.1 Sex

Variable: sex

Type: Binary, [male=0, female=1]

Description. We obtained information on participants’ sex from registries administered by the Digital and Population Data Services Agency.

2.2.2 Age

Variable: age_cont

Type: Continuous, [natural number]

Description. We obtained information on participants’ age from registries administered by the Digital and Population Data Services Agency.

2.2.3 Marital status

Variable: marital_status

Type: Factor, [“married, in a registered relationship or cohabiting”, “separated, divorced, widowed or single”]

Description. All surveys had an identical question on marital status. Responders were asked “Are you currently: married or in a registered relationship, cohabiting, separated or divorced, widowed, single”. We created a categorical variable with two categories.

Questions, answers and equivalence are shown below:

| **Survey** | **Question** | **Answer** | **Equivalence** |
| --- | --- | --- | --- |
| FinSote 2018 | 1. Are you currently: | 1. Married or in a registered relationship  2. cohabiting  3. separated or divorced  4. widowed  5. single | Married, in a registered relationship or cohabiting 1 or 2  Separated, divorced, widowed or single 3, 4 or 5 |
| FinSote 2019 | 1. Are you currently: | 1. Married or in a registered relationship  2. cohabiting  3. separated or divorced  4. widowed  5. single | Married, in a registered relationship or cohabiting 1 or 2  Separated, divorced, widowed or single 3, 4 or 5 |
| FinSote 2020 | 3. Are you currently: | 1. Married or in a registered relationship  2. cohabiting  3. separated or divorced  4. widowed  5. single | Married, in a registered relationship or cohabiting 1 or 2  Separated, divorced, widowed or single 3, 4 or 5 |

2.2.4 Years of education

Variable:educ_years

Type: Continuous, [natural number]

Description. All three surveys had identical questions on the number of years of full time studies.

Questions, answers and equivalence are shown below:

| **Survey** | **Question** | **Answer** |
| --- | --- | --- |
| FinSote 2018 | 2. How many years altogether have you attended school or studied full time? Including primary and comprehensive school. | _ years |
| FinSote 2019 | 4. How many years altogether have you attended school or studied full time? Including primary and comprehensive school. | _ years |
| FinSote 2020 | 4. How many years altogether have you attended school or studied full time? Including primary and comprehensive school. | _ years |

2.2.5 Mother tongue

Variable: mother_tongue

Type: Factor, [“Finnish”, “Swedish” and “others”]

Description. We obtained information on participants’ mother tongue from registries administered by the Digital and Population Data Services Agency.

2.2.7 Participation in social activities

Variable: involvement_attend_j

Type: Factor, [“no participation”, “occasional”, “active”]

Description. All three surveys had an identical question on participation in social activities.

Questions, answers and equivalence are shown below:

| **Survey** | **Question** | **Answer** | **Equivalence** |
| --- | --- | --- | --- |
| FinSote 2018 | 11. Do you participate in the activities of any club, association, hobby group or religious or spiritual community (e.g. a sports club, residents’ association, political party, choir, parish)? | 1.no  2. yes, actively  3. Yes, occasionally | No participation 1  Occasional 3  Active 2 |
| FinSote 2019 | 89. Do you participate in the activities of any club, association, hobby group or religious or spiritual community (e.g. a sports club, residents’ association, political party, choir, parish)? | 1.no  2. yes, actively  3. Yes, occasionally | No participation 1  Occasional 3  Active 2 |
| FinSote 2020 | 12. Do you participate in the activities of any club, association, hobby group or religious or spiritual community (e.g. a sports club, residents’ association, political party, choir, parish)? | 1.no  2. yes, actively  3. Yes, occasionally | No participation 1  Occasional 3  Active 2 |

## **2.3 Potential collider**

2.3.1 Body mass index

Variable: bmi

Type: Continuous, [natural number]

Description. All three surveys had an identical question on height and weight. Height was assessed with a question “How tall are you?” and weight with a question “ How much do you weigh when wearing light clothing?”. We handled extreme values by setting a cut-off using data from Finrisk 2012, which is a health examination survey with standardized measurement techniques. We, therefore, consider a height below 137 cms and above 218 cms as an extreme outlier and coded as a missing value.

Questions, answers and equivalence are shown below:

| **Survey** | **Question** | **Answer** | **Equivalence** |
| --- | --- | --- | --- |
| FinSote 2018 | 22. How tall are you? ________ cm please round to nearest centimetre  23. How much do you weigh when wearing light clothing? ________ kg please round to nearest kilogramme | _ cm  _kg | BMI calculated as kg/m^2^ |
| FinSote 2018 | 56. How tall are you? ________ cm please round to nearest centimetre  57. How much do you weigh when wearing light clothing? ________ kg please round to nearest kilogramme | _ cm  _kg | BMI calculated as kg/m^2^ |
| FinSote 2018 | 20. How tall are you? ________ cm please round to nearest centimeter  21. How much do you weigh when wearing light clothing? ________ kg please round to nearest kilogramme | _ cm  _kg | BMI calculated as kg/m^2^ |

# Missing data in FinSote 2018-2020

## **Table S1**. Proportion of missing data by variables and survey year

|  | **2018** | | **2019** | | **2020** | |
| --- | --- | --- | --- | --- | --- | --- |
|  | **n** | **%** | **n** | **%** | **n** | **%** |
| Analytical sample | 14736 | 100 | 6251 | 100 | 28199 | 100 |
| Smoking status | 502 | 3.4 | 137 | 2.2 | 799 | 2.8 |
| Sex | 0 | 0 | 0 | 0 | 0 | 0 |
| Age | 0 | 0 | 0 | 0 | 0 | 0 |
| Hospital district | 0 | 0 | 0 | 0 | 0 | 0 |
| Marital status | 94 | 0.6 | 64 | 1.0 | 436 | 1.5 |
| Years of education | 404 | 2.7 | 198 | 3.2 | 1303 | 4.6 |
| Mother tongue | 0 | 0 | 0 | 0 | 0 | 0 |
| Social participation | 189 | 1.3 | 125 | 2.0 | 520 | 1.8 |
| Body mass index | 284 | 1.9 | 170 | 2.7 | 900 | 3.2 |

# Baseline characteristics of FinSote participants by other forms of tobacco use

## **Table S2**. Baseline characteristics of 32931 FinSote participants by snus use

|  | **Never user** | **Former user** | **Current user** |
| --- | --- | --- | --- |
| n | 28276 | 3628 | 1027 |
| confirmed COVID-19 cases | 292 | 47 | 30 |
| Sex, % female | 56.9 | 29.0 | 10.7 |
| Mean age, (SD) | 48.2 (16.1) | 42.6 (14.8) | 33.1 (10.3) |
| Marital status, % separated, single or widowed | 32.5 | 33.0 | 43.4 |
| Mean years of education (SD) | 14.3 (3.9) | 14.4 (4.0) | 14.5 (3.3) |
| Mother tongue, % |  |  |  |
| Finnish | 94 | 91.0 | 88.4 |
| Swedish | 4 | 7.4 | 10 |
| Other | 2.1 | 1.6 | 1.6 |
| Participation in social activities, % |  |  |  |
| No participation | 49.7 | 52.3 | 52.3 |
| Active | 27.1 | 25.5 | 31.1 |
| Occasional | 23.2 | 22.2 | 16.6 |
| Mean BMI (SD) | 26.8 (5.4) | 26.9 (5.0) | 26.6 (4.6) |

Data are means (standard deviation) and percentages. Means and percentages incorporate complex survey design

## **Table S3**. Baseline characteristics of 27098 FinSote participants by e-cigarette with nicotine use

|  | **Never user** | **Former user** | **Current user** |
| --- | --- | --- | --- |
| n | 23542 | 3236 | 320 |
| confirmed COVID-19 cases | 231 | 42 | 4 |
| Sex, % female | 54.1 | 32.8 | 31.6 |
| Mean age, (SD) | 47.3 (15.4) | 41.4 (15.3) | 38.1 (14.0) |
| Marital status, % separated, single or widowed | 31.4 | 37.1 | 45.5 |
| Mean years of education (SD) | 14.5 (3.7) | 13.9 (3.5) | 13.7 (3.1) |
| Mother tongue, % |  |  |  |
| Finnish | 93.5 | 91.5 | 89.9 |
| Swedish | 4.4 | 5.5 | 8 |
| Other | 2.1 | 3.0 | 2.1 |
| Participation in social activities, % |  |  |  |
| No participation | 49.1 | 58.1 | 71.4 |
| Active | 27.9 | 22.3 | 16.4 |
| Occasional | 23 | 19.5 | 12.2 |
| Mean BMI (SD) | 26.8 (5.4) | 27.1 (5.2) | 27.8 (6.3) |
|  |  |  |  |

Data are means (standard deviation) and percentages. Means and percentages incorporate complex survey design

## **Table S4**. Baseline characteristics of 27005 FinSote participants by e-cigarette without nicotine use

|  | **Never user** | **Former user** | **Current user** |
| --- | --- | --- | --- |
| n | 23931 | 2966 | 108 |
| confirmed COVID-19 cases | 233 | 43 | 1 |
| Sex, % female | 53.5 | 32.90 | 28.1 |
| Mean age, (SD) | 47.1 (15.4) | 41.7 (15.4) | 36.5 (14.1) |
| Marital status, % separated, single or widowed | 31.5 | 37.50 | 53.6 |
| Mean years of education (SD) | 14.5 (3.7) | 13.9 (3.4) | 13.1 (3.8) |
| Mother tongue, % |  |  |  |
| Finnish | 93.5 | 91.20 | 87.3 |
| Swedish | 4.4 | 5.60 | 10.9 |
| Other | 2.1 | 3.20 | 1.8 |
| Participation in social activities, % |  |  |  |
| No participation | 49.3 | 58.50 | 77.4 |
| Active | 27.9 | 21.90 | 11.7 |
| Occasional | 22.8 | 19.70 | 11 |
| Mean BMI (SD) | 26.8 (5.4) | 27.1 (5.3) | 28.8 (6.6) |
|  |  |  |  |

Data are means (standard deviation) and percentages. Means and percentages incorporate complex survey design

## **Table S5**. Baseline characteristics of 27073 FinSote participants by nicotine replacement product use

|  | **Never user** | **Former user** | **Current user** |
| --- | --- | --- | --- |
| n | 22040 | 3814 | 1219 |
| confirmed COVID-19 cases | 210 | 48 | 16 |
| Sex, % female | 54.3 | 35.9 | 39 |
| Mean age, (SD) | 46.9 (15.8) | 43.9 (14.7) | 45.8 (13.6) |
| Marital status, % separated, single or widowed | 31.9 | 32.7 | 39.7 |
| Mean years of education (SD) | 14.5 (3.7) | 13.9 (3.5) | 14.0 (3.5) |
| Mother tongue, % |  |  |  |
| Finnish | 93.4 | 91.5 | 94.4 |
| Swedish | 4.5 | 5.4 | 4.8 |
| Other | 2.1 | 3.0 | 0.8 |
| Participation in social activities, % |  |  |  |
| No participation | 48.8 | 57.1 | 61 |
| Active | 28.3 | 22.6 | 18.1 |
| Occasional | 22.9 | 20.2 | 21 |
| Mean BMI (SD) | 26.7 (5.3) | 27.6 (5.6) | 26.9 (4.9) |

Data are means (standard deviation) and percentages. Means and percentages incorporate complex survey design

# Sensitivity analyses using data from FinSote 2018 and 2019

**Sensitivity analysis (i)**. We conducted the same analyses but restricted the data to FinSote 2018 and 2019 (Table S6).

## **Table S6**. Relative risk of confirmed COVID-19 cases by tobacco use in participants of FinSote surveys 2018 and 2019

|  | **COVID-19 cases** | **Relative risk (95% CI)** | | |
| --- | --- | --- | --- | --- |
|  |  | Model 1: Adjusted for age and sex | Model 2: Model 1 and all confounders | Model 3: Model 2 and potential collider |
| **Smoking status (n=19102)** | | | | |
| Daily smoker | 17 | 1.52 (0.69; 3.35) | 1.28 (0.59; 2.8) | 1.27 (0.61; 2.64) |
| Occasional smoker | 11 | 0.61 (0.3; 1.24) | 0.62 (0.3; 1.3) | 0.57 (0.27; 1.19) |
| Former smoker | 67 | 1.21 (0.71; 2.06) | 1.11 (0.69; 1.78) | 1.09 (0.68; 1.75) |
| Never smoker | 89 | ref | ref | ref |
|  |  |  |  |  |
| **Snus use (n=15974)** |  |  |  |  |
| Current user | 13 | 1.13 (0.54; 2.37) | 1.2 (0.57; 2.57) | 1.25 (0.57; 2.73) |
| Former user | 21 | 0.95 (0.41; 2.19) | 0.98 (0.47; 2.08) | 0.98 (0.47; 2.07) |
| Never user | 145 | ref | ref | ref |
|  |  |  |  |  |
| **E-cigarette with nicotine (n=10181)** | | | | |
| Current user | 1 | 2.35 (0.31; 17.93) | 2.85 (0.39; 20.86) | 1.95 (0.52; 7.25) |
| Former user | 15 | 1.16 (0.44; 3.03) | 0.97 (0.4; 2.37) | 1.03 (0.41; 2.6) |
| Never user | 71 | ref | ref | ref |
|  |  |  |  |  |
| **E-cigarette without nicotine (n=10155)** | | | | |
| Current user | 0 | - | - | - |
| Former user | 16 | 1.61 (0.66; 3.94) | 1.37 (0.57; 3.31) | 1.32 (0.55; 3.14) |
| Never user | 71 | ref | ref | ref |
|  |  |  |  |  |
| **Nicotine replacement products (n=10187)** | | | | |
| Current user | 4 | 1.64 (0.36; 7.42) | 1.82 (0.43; 7.63) | 1.72 (0.51; 5.86) |
| Former user | 17 | 1.1 (0.43; 2.82) | 0.99 (0.42; 2.35) | 1 (0.41; 2.45) |
| Never user | 66 | ref | ref | ref |

Estimates for smoking include all age groups and all Finsote surveys. Estimates on snus use include participants 20-74 years old in FinSote 2018 and all age groups in FinSote 2019. Estimates on e-cigarettes and nicotine replacement products include all age groups in FinSote 2018.

Model 2 adjusted for marital status, years of education, mother tongue, and participation in social activities. Model 3 adjusted additionally for BMI. Analyses incorporate complex sampling design.

**Sensitivity analysis (ii)**. We conducted the same analyses as (i) but using Cox proportional hazards models. We tested the proportional hazards assumption by plotting Schoenfeld residuals against time.

Models 3 and 4 run into singular fit problems and thus, to obtain a coefficient, we modelled the variables as linear predictors instead of using penalised smoothing splines. In the case of the models with e-cigarettes with and without nicotine and nicotine replacement therapy, we also had to remove the fixed effects for hospital districts for the same reason.

We tested the proportional hazards assumption by plotting Schoenfeld residuals against time. The results show that the continuous variables mildly violate the proportional hazards function, as their effect seems to slightly decrease with time. This indicates, again, that the models do not fit well, as there are very few events in the data. The obtained coefficients, anyhow, seem to be reasonably similar to the ones obtained using a Poisson regression.

## **Table S7**. Hazard ratios of confirmed COVID-19 cases by tobacco use in participants of FinSote surveys 2018 and 2019

|  | **COVID-19 cases** | **Hazard ratio (95% CI)** | | |
| --- | --- | --- | --- | --- |
|  |  | Model 1: Adjusted for age and sex | Model 2: Model 1 and all confounders | Model 3: Model 2 and potential collider |
| **Smoking status (n=19243)** | | | | |
| Daily smoker | 17 | 1.5 (0.67; 3.35) | 1.33 (0.6; 2.97) | 1.3 (0.59; 2.89) |
| Occasional smoker | 11 | 0.6 (0.29; 1.23) | 0.62 (0.29; 1.29) | 0.6 (0.29; 1.26) |
| Former smoker | 67 | 1.21 (0.71; 2.08) | 1.14 (0.69; 1.89) | 1.13 (0.68; 1.86) |
| Never smoker | 89 | ref | ref | ref |
|  |  |  |  |  |
| **Snus use (n=15974)** |  |  |  |  |
| Current user | 13 | 1.04 (0.5; 2.19) | 1.02 (0.44; 2.38) | 1.05 (0.45; 2.43) |
| Former user | 21 | 0.9 (0.39; 2.08) | 0.96 (0.44; 2.11) | 0.96 (0.44; 2.1) |
| Never user | 145 | ref | ref | ref |
|  |  |  |  |  |
| **E-cigarette with nicotine (n=10181)** | | | | |
| Current user | 1 | 2.22 (0.27; 18.07) | 2.19 (0.27; 17.68) | 2.09 (0.27; 16.02) |
| Former user | 15 | 1.09 (0.42; 2.82) | 0.91 (0.35; 2.39) | 0.9 (0.34; 2.37) |
| Never user | 71 | ref | ref | ref |
|  |  |  |  |  |
| **E-cigarette without nicotine (n=10155)** | | | | |
| Current user | 0 | - | - | - |
| Former user | 16 | 1.63 (0.66; 4.04) | 1.36 (0.52; 3.54) | 1.35 (0.53; 3.46) |
| Never user | 71 | ref | ref | ref |
|  |  |  |  |  |
| **Nicotine replacement products (n=10187)** | | | | |
| Current user | 4 | 1.68 (0.36; 7.81) | 1.84 (0.4; 8.45) | 1.81 (0.4; 8.18) |
| Former user | 17 | 1.11 (0.43; 2.84) | 1.02 (0.41; 2.56) | 0.98 (0.37; 2.55) |
| Never user | 66 | ref | ref | ref |

Estimates for smoking include all age groups and all Finsote surveys. Estimates on snus use include participants 20-74 years old in FinSote 2018 and all age groups in FinSote 2019. Estimates on e-cigarettes and nicotine replacement products include all age groups in FinSote 2018.

Model 2 adjusted for marital status, years of education, mother tongue, and participation in social activities. Model 3 adjusted additionally for BMI. Analyses incorporate complex sampling design.

**Sensitivity analysis (iii)**. We re-conducted the main analyses but excluding current users of any other form of tobacco, respectively. For example, in the models with smoking status as the exposure, we excluded current users of snus, e-cigarettes with nicotine and nicotine replacement therapy products.

We tested two different approaches. In Table S8, we show the results excluding other current users of tobacco but did not exclude those with missing data on the tobacco variables. Given that data on other forms of tobacco use are not available for all participants, this results in a small reduction of the sample size. In Table S9, we excluded other current users of tobacco but also those with missing data on the tobacco variables. This results in a much smaller sample size.

##

## **Table S8**. Relative risk of confirmed COVID-19 cases by tobacco use in participants of FinSote surveys after excluding other tobacco users

|  | **COVID-19 cases** | **Relative risk (95% CI)** | | |
| --- | --- | --- | --- | --- |
|  |  | Model 1: Adjusted for age and sex | Model 2: Model 1 and all confounders | Model 3: Model 2 and potential collider |
| **Smoking status (n=41948)** | | | | |
| Daily smoker | 25 | 1.02 (0.52; 2) | 1.02 (0.53; 1.94) | 1.01 (0.53; 1.93) |
| Occasional smoker | 20 | 0.73 (0.39; 1.36) | 0.73 (0.38; 1.38) | 0.71 (0.38; 1.36) |
| Former smoker | 138 | 1 (0.71; 1.41) | 1 (0.72; 1.39) | 1 (0.72; 1.38) |
| Never smoker | 171 | ref | ref | ref |
|  |  |  |  |  |
| **Snus use (n=27113)** |  |  |  |  |
| Current user | 16 | 2.19 (1.14; 4.21) | 2.22 (1.13; 4.35) | 2.16 (1.1; 4.25) |
| Former user | 34 | 0.92 (0.57; 1.49) | 0.97 (0.6; 1.55) | 0.95 (0.59; 1.52) |
| Never user | 250 | ref | ref | ref |
|  |  |  |  |  |
| **E-cigarette with nicotine (n=21765)** | | | | |
| Current user | 0 | - | - | - |
| Former user | 23 | 0.86 (0.5; 1.48) | 0.83 (0.47; 1.47) | 0.81 (0.45; 1.43) |
| Never user | 191 | ref | ref | ref |
|  |  |  |  |  |
| **E-cigarette without nicotine (n=21665)** | | | | |
| Current user | 0 | - | - | - |
| Former user | 20 | 0.88 (0.5; 1.55) | 0.84 (0.46; 1.54) | 0.82 (0.45; 1.5) |
| Never user | 194 | ref | ref | ref |
|  |  |  |  |  |
| **Nicotine replacement products (n=21943)** | | | | |
| Current user | 5 | 2.01 (0.74; 5.48) | 2.24 (0.82; 6.1) | 2.27 (0.83; 6.22) |
| Former user | 28 | 0.91 (0.55; 1.51) | 0.9 (0.53; 1.54) | 0.91 (0.54; 1.53) |
| Never user | 182 | ref | ref | ref |

Estimates for smoking include all age groups and all Finsote surveys. Estimates on snus use include participants 20-74 years old in FinSote 2018 and 2020 and all age groups in FinSote 2019. Estimates on e-cigarettes and nicotine replacement products include all age groups in FinSote 2018 and 2020.

Model 2 adjusted for marital status, years of education, mother tongue, and participation in social activities. Model 3 adjusted additionally for BMI. Analyses incorporate complex sampling design.

## **Table S9**. Relative risk of confirmed COVID-19 cases by tobacco use in participants of FinSote surveys after excluding other tobacco users and those with missing data on tobacco products

|  | **COVID-19 cases** | **Relative risk (95% CI)** | | |
| --- | --- | --- | --- | --- |
|  |  | Model 1: Adjusted for age and sex | Model 2: Model 1 and all confounders | Model 3: Model 2 and potential collider |
| **Smoking status (n=24666)** | | | | |
| Daily smoker | 17 | 1.18 (0.55; 2.55) | 1.14 (0.53; 2.43) | 1.13 (0.53; 2.42) |
| Occasional smoker | 13 | 0.72 (0.33; 1.57) | 0.71 (0.31; 1.59) | 0.7 (0.31; 1.57) |
| Former smoker | 108 | 1.14 (0.77; 1.7) | 1.14 (0.79; 1.65) | 1.14 (0.79; 1.65) |
| Never smoker | 97 | ref | ref | ref |
|  |  |  |  |  |
| **Snus use (n=21689)** |  |  |  |  |
| Current user | 11 | 2.28 (1.05; 4.95) | 2.27 (1.02; 5.07) | 2.22 (0.99; 4.94) |
| Former user | 26 | 0.89 (0.51; 1.56) | 0.95 (0.55; 1.63) | 0.93 (0.54; 1.6) |
| Never user | 179 | ref | ref | ref |
|  |  |  |  |  |
| **E-cigarette with nicotine (n=21352)** | | | | |
| Current user | 0 | - | - | - |
| Former user | 21 | 0.87 (0.5; 1.53) | 0.85 (0.47; 1.54) | 0.83 (0.46; ) |
| Never user | 184 | ref | ref | ref |
|  |  |  |  |  |
| **E-cigarette without nicotine (n=21232)** | | | | |
| Current user | 0 | 0 (0; 0) | 0 (0; 0) | 0 (0; 0) |
| Former user | 19 | 0.9 (0.5; 1.61) | 0.88 (0.47; 1.64) | 0.86 (0.47; 1.6) |
| Never user | 186 | ref | ref | ref |
|  |  |  |  |  |
| **Nicotine replacement products (n=21557)** | | | | |
| Current user | 5 | 2.38 (0.87; 6.48) | 2.73 (1; 7.48) | 2.82 (1.02; 7.77) |
| Former user | 28 | 0.96 (0.58; 1.59) | 0.95 (0.56; 1.63) | 0.96 (0.57; 1.62) |
| Never user | 177 | ref | ref | ref |

Estimates for smoking include all age groups and all Finsote surveys. Estimates on snus use include participants 20-74 years old in FinSote 2018 and 2020 and all age groups in FinSote 2019. Estimates on e-cigarettes and nicotine replacement products include all age groups in FinSote 2018 and 2020.

Model 2 adjusted for marital status, years of education, mother tongue, and participation in social activities. Model 3 adjusted additionally for BMI. Analyses incorporate complex sampling design.

## **Table S10**. Relative risk of confirmed COVID-19 cases by tobacco use in participants of FinSote surveys by time periods (before and after the start of COVID-19 vaccination rollout)

|  | **Pre-vaccination period (Feb 26-Dec 26, 2020)** | | | **Post-vaccination period (Dec 27, 2020 - Aug 23, 2021)** | | |
| --- | --- | --- | --- | --- | --- | --- |
|  | **COVID-19 cases** | **Relative risk (95% CI)** | | **COVID-19 cases** | **Relative risk (95% CI)** | |
|  |  | Model 1: Adjusted for age and sex | Model 2: Model 1 and all confounders |  | Model 1: Adjusted for age and sex | Model 2: Model 1 and all confounders |
| **Smoking status (n=44199)** |  |  |  |  |  |  |
| Current daily smoker | 6 | 0.24 (0.09; 0.64) | 0.27 (0.1; 0.74) | 28 | 1.64 (0.87; 3.07) | 1.52 (0.83; 2.79) |
| Current occasional smoker | 6 | 0.47 (0.12; 1.79) | 0.48 (0.12; 1.88) | 23 | 0.85 (0.5; 1.45) | 0.87 (0.51; 1.48) |
| Former smoker | 66 | 1.44 (0.86; 2.41) | 1.48 (0.9; 2.44) | 90 | 0.83 (0.56; 1.22) | 0.82 (0.55; 1.22) |
| Never smoker | 74 | ref | ref | 102 | ref | ref |
|  |  |  |  |  |  |  |
| **Snus use (n=32931)** |  |  |  |  |  |  |
| Current user | 10 | 2.01 (0.8; 5.08) | 2.18 (0.87; 5.49) | 20 | 1.38 (0.78; 2.42) | 1.46 (0.83; 2.57) |
| Former user | 14 | 0.59 (0.27; 1.26) | 0.64 (0.31; 1.32) | 33 | 1.24 (0.71; 2.17) | 1.33 (0.79; 2.25) |
| Never user | 116 | ref | ref | 176 | ref | ref |

Estimates for smoking include all age groups and all Finsote surveys. Estimates on snus use include participants 20-74 years old in FinSote 2018 and 2020 and all age groups in FinSote 2019. Model 2 adjusted for marital status, years of education, mother tongue, and participation in social activities.
