# Supplementary material for: Tobacco use and risk of COVID-19 infection in the Finnish general population
Source: Sci Rep. 2022 Nov 25;12:20335. doi: 10.1038/s41598-022-24148-y (PMC9700668; doi:10.1038/s41598-022-24148-y)
Supplement: Supplementary file 2 — Supplementary Information 2. [file 41598_2022_24148_MOESM2_ESM.pdf]

Statistical code - Tobacco use and risk of COVID-19 infection in the Finnish general population

Sebastián Peña et al

Sept 30 2022

Loading packages and dataset

```
library(readxl)
library(haven)
library(styler)
library(tidyverse)
library(knitr)
library(kableExtra)
library(tidyr)
library(janitor)
library(gtools)
library(ggridges)
library(ggfortify)
library(tableone)
library(pollster)
library(naniar)
library(survey)
library(survival)
library(biostat3)
library(Epi)

options(scipen=999)

# Loading FinSote dataset
finsote2018 <- read_sas("//helfs01.thl.fi/groups4/Tobrisk_FinSote/Data and code/FinSote2018/data_f181.sa
finsote2019 <- read_sas("//helfs01.thl.fi/groups4/Tobrisk_FinSote/Data and code/FinSote2019/data_f191.sa
finsote2020 <- read_sas("//helfs01.thl.fi/groups4/Tobrisk_FinSote/Data and code/FinSote2020/data_f201.sa
finsote2020_dates <- read_excel("//helfs01.thl.fi/groups4/Tobrisk_FinSote/Data and code/FinSote2020/F20
finsote2020covid <- read_sas("//helfs01.thl.fi/groups4/Tobrisk_FinSote/Data and code/FinSote2020_covid/c
ttr <- read_excel("//helfs01.thl.fi/groups4/Tobrisk_FinSote/Data and code/TTR/ttr_covid_f201_f191_f181.x
ttr2 <- read_excel("//helfs01.thl.fi/groups4/Tobrisk_FinSote/Data and code/TTR/ttr_covid_f201_f191_f181_
```

Management of variables

We harmonised all three datasets according to the variables described in the study protocol. FinSote 2018 and 2020 have identical questions for the variables of interest; we, therefore, created a unique dataset

appending both datasets. FinSote 2019 was conducted in conjunction with EHIS 3 and questions are not always identical but can produce harmonized variables. This is particularly true for smoking questions. The code will note when questions are identical or different, more information can be found in the Supplementary Appendix.

The section creates a pooled dataset (which includes participants with missing data) of 49186 participants, including all participants in 2018, 2019 and 2020 who provided consent for data linkage.

```
# FINSOTE 2018 and 2020
# Creating data id FinSote 2018 and subset with key variables
finsote2018_brief <- finsote2018 %>%
  dplyr::select(ID, GUMM85ID, consent_reg, maritalstatus, educ_years, IKA2, height_cm,
                weight_kg, involvement_attend_j, smoke_cur_never, smoke_altprod_snus,
                smoke_altprod_nic_ecig, smoke_altprod_non_nic_ecig,
                smoke_altprod_nic_substitute, smoke_altprod_recipe, smoke_altprod_other,
                Sukupuoli, kieliryhma, fs_shp_koodi, fs_shp_nimi_fi, rg_N1, w_analysis1,
                rg_stratum1, w_expansion1) %>%
  rename(rg_N_suomi=rg_N1) %>%
  rename(w_analysis_suomi=w_analysis1) %>%
  rename(rg_stratum_suomi=rg_stratum1) %>%
  rename(w_expansion_suomi=w_expansion1) %>%
  mutate(dataid=2018) %>%
  mutate(age_cont=IKA2) %>%
  filter(consent_reg==1) ##This excludes participants who didn't agree on the linkage

# Appending datasets with different data types creates problems
finsote2018_brief$Sukupuoli <- as.factor(finsote2018_brief$Sukupuoli)

# Creating data id FinSote 2020 and subset with key variables
finsote2020_brief <- finsote2020 %>%
  dplyr::select(ID, GUMM85ID, maritalstatus, educ_years, IKA2, height_cm, weight_kg,
                involvement_attend_j, smoke_cur_never, smoke_altprod_snus,
                smoke_altprod_nic_ecig, smoke_altprod_non_nic_ecig,
                smoke_altprod_nic_substitute, sukupuoli, kieliryhma, fs_shp_koodi,
                rg_N_suomi, w_analysis_suomi,
                rg_stratum_suomi, w_expansion_suomi) %>%
  rename(Sukupuoli=sukupuoli) %>%
  mutate(dataid=2020) %>%
  mutate(age_cont=IKA2)

# Appending datasets with different data types creates problems
finsote2020_brief$Sukupuoli <- as.factor(finsote2020_brief$Sukupuoli)

# Appending both datasets
finsote1820 <- bind_rows(finsote2018_brief, finsote2020_brief)  #dplyr works much
#better than smartbind
miss_var_summary(finsote1820)                                   #now looks good

finsote1820 <- finsote1820 %>%
  mutate(mother_tongue = kieliryhma) %>%
  mutate(sex=Sukupuoli)

# Checking class and levels of key variables
class(finsote1820$smoke_cur_never)  #numeric
class(finsote1820$maritalstatus)    #numeric
```

```r
class(finsote1820$educ_years)       #numeric. ready for use
class(finsote1820$sex)              #factor
class(finsote1820$mother_tongue)    #numeric

# Tobacco use. Convert to factor, creating levels
## Smoking
finsote1820$smoking_status <- as.factor(finsote1820$smoke_cur_never)
finsote1820$smoking_status <-  recode_factor(finsote1820$smoking_status,
                                             `1`="daily smoker",
                                             `2`="occasional",
                                             `3`="former smoker",
                                             `4`="never smoker")

## Snus
finsote1820$snus_status <- as.factor(finsote1820$smoke_altprod_snus)
finsote1820$snus_status <-  recode_factor(finsote1820$snus_status,
                                          `1`="daily user",
                                          `2`="occasional",
                                          `3`="former user",
                                          `4`="never user")

## E-cig with nicotine
finsote1820$ecig_nic_status <- as.factor(finsote1820$smoke_altprod_nic_ecig)
finsote1820$ecig_nic_status <-  recode_factor(finsote1820$ecig_nic_status,
                                              `1`="daily user",
                                              `2`="occasional",
                                              `3`="former user",
                                              `4`="never user")

## E-cig without nicotine
finsote1820$ecig_nonic_status <- as.factor(finsote1820$smoke_altprod_non_nic_ecig)
finsote1820$ecig_nonic_status <-  recode_factor(finsote1820$ecig_nonic_status,
                                                `1`="daily user",
                                                `2`="occasional",
                                                `3`="former user",
                                                `4`="never user")

## Nicotine replacement therapy
finsote1820$nrt_status <- as.factor(finsote1820$smoke_altprod_nic_substitute)
finsote1820$nrt_status <-  recode_factor(finsote1820$nrt_status,
                                         `1`="daily user",
                                         `2`="occasional",
                                         `3`="former user",
                                         `4`="never user")

# Confounders
## Marital status. Convert to factor, create categorical levels
finsote1820$maritalstatus_bin <- as.factor(finsote1820$maritalstatus)
finsote1820$maritalstatus_bin <-  recode_factor(finsote1820$maritalstatus_bin,
                                                `1`="married or cohabiting",
                                                `2`="married or cohabiting",
                                                `3`="separated, single or widowed",
                                                `4`="separated, single or widowed",
```

```
                                                `5`="separated, single or widowed")

## Mother tongue
finsote1820$mother_tongue <- as.factor(finsote1820$mother_tongue)
finsote1820$mother_tongue <-  recode_factor(finsote1820$mother_tongue,
                                            `1`="finnish",
                                            `2`="swedish",
                                            `3`="other",
                                            `4`="other")

## Sex. Needs no modification. 1==male and 2==female
##Years of education. Needs no modification, continuous variable.

## Social participation
finsote1820$involvement_attend_j <- as.factor(finsote1820$involvement_attend_j)
finsote1820$involvement_attend_j <-  recode_factor(finsote1820$involvement_attend_j,
                                                   `1`="no participation",
                                                   `2`="active",
                                                   `3`="occasional")

# Potential collider
## BMI
class(finsote1820$height_cm) #with the merge, both variables convert to character
class(finsote1820$weight_kg) #with the merge, both variables convert to character
finsote1820$height_cm <- as.numeric(finsote1820$height_cm)
finsote1820$weight_kg <- as.numeric(finsote1820$weight_kg)
finsote1820 <- finsote1820 %>%
  mutate(bmi = weight_kg/(height_cm*height_cm/10000))

# Convert complex survey variables
finsote1820$rg_N_suomi <- as.numeric(finsote1820$rg_N_suomi)
finsote1820$rg_stratum_suomi <- as.numeric(finsote1820$rg_stratum_suomi)
finsote1820$w_analysis_suomi <- as.numeric(finsote1820$w_analysis_suomi)
finsote1820$w_expansion_suomi <- as.numeric(finsote1820$w_expansion_suomi)
miss_var_summary(finsote2020_brief)

# Check variable types
class(finsote1820$maritalstatus_bin) #factor
class(finsote1820$sex) #factor
class(finsote1820$rg_N_suomi) #numeric

# Dataset combining FinSote 2018 and 2020 ready

# FINSOTE 2019
## Creating data id FinSote 2020 and subset with key variables
finsote2019_brief <- finsote2019 %>%
  dplyr::select(ID, GUMM85ID, maritalstatus, educ_years, IKA2, height_cm,
                weight_kg, involvement_attend_j, ehis_smoking_current,
                ehis_smoking_product, ehis_smoking_cigarettes,  smoke_oneyear,
                smoke_years,    ehis_smoke_indoor,
                smoke_snus_now2,    smoke_ecig_now, Sukupuoli, kieliryhma, fs_shp_koodi,
                fs_shp_nimi_fi, rg_N, rg_stratum, w_analysis, w_expansion) %>%
  rename(rg_N_suomi=rg_N) %>%
```

```
  rename(w_analysis_suomi=w_analysis) %>%
  rename(rg_stratum_suomi=rg_stratum) %>%
  rename(w_expansion_suomi=w_expansion) %>%
  mutate(age_cont=IKA2) %>%
  mutate(dataid=2019)

finsote2019_brief <- finsote2019_brief %>%
  mutate(mother_tongue = kieliryhma) %>%
  mutate(sex=Sukupuoli)

# Checking class and levels of key variables
class(finsote2019_brief$maritalstatus)    #numeric
class(finsote2019_brief$educ_years)       #numeric. ready for use
class(finsote2019_brief$sex)              #character
class(finsote2019_brief$mother_tongue)    #numeric

## All variables are identical to FinSote 2018 and 2020 except for smoking
class(finsote2019_brief$ehis_smoking_current)
class(finsote2019_brief$ehis_smoking_product)
class(finsote2019_brief$smoke_oneyear)

class(finsote2019_brief$smoke_snus_now2)
class(finsote2019_brief$smoke_ecig_now)
table(finsote2019_brief$ehis_smoking_current, useNA = "ifany")
table(finsote2019_brief$ehis_smoking_current, finsote2019_brief$smoke_oneyear,
      useNA = "ifany")
table(finsote2019_brief$smoke_oneyear, useNA = "ifany")

## Smoking (see Supplementary Appendix for more details)
### With question 84, we can break down the "not at all" group into
#never and former smokers
finsote2019_brief <- finsote2019_brief %>%
  mutate(smoking_status=ehis_smoking_current) %>%
  mutate(smoking_status=replace(smoking_status, smoking_status==3 &
                                  smoke_oneyear==1, 5))   %>%
  #replaces with "former smoker"
  mutate(smoking_status=replace(smoking_status, smoking_status==3 &
                                  smoke_oneyear==2, 4))
#replaces with "never smoker" %>%

finsote2019_brief <- finsote2019_brief %>%
  mutate(smoking_status=replace(smoking_status, smoking_status==3, NA))
#replaces with NA those who answered 3 in question 81 but not answered 84

### Quality checks
table(finsote2019_brief$ehis_smoking_current, finsote2019_brief$smoke_oneyear)
table(finsote2019_brief$smoking_status, finsote2019_brief$smoke_oneyear,
      useNA = "ifany") #looks good
table(finsote2019_brief$smoking_status)

### Converting into factor and labeling
finsote2019_brief$smoking_status <- as.factor(finsote2019_brief$smoking_status)
finsote2019_brief$smoking_status <-  recode_factor(finsote2019_brief$smoking_status,
```

```
                                                   `1`="daily smoker",
                                                   `2`="occasional",
                                                   `4`="former smoker",
                                                   `5`="never smoker")

### Quality checks
table(finsote2019_brief$smoking_status, finsote2019_brief$ehis_smoking_current,
      useNA = "ifany")
table(finsote2019_brief$smoking_status, finsote2019_brief$smoke_oneyear,
      useNA = "ifany") #looks good

### Snus and e-cigarettes
finsote2019_brief$snus_status <- as.factor(finsote2019_brief$smoke_snus_now2)
finsote2019_brief$snus_status <-  recode_factor(finsote2019_brief$snus_status,
                                                `1`="daily user",
                                                `2`="occasional",
                                                `3`="former user",
                                                `4`="never user")

finsote2019_brief$ecig_status <- as.factor(finsote2019_brief$smoke_ecig_now)
finsote2019_brief$ecig_status <-  recode_factor(finsote2019_brief$ecig_status,
                                                `1`="daily smoker",
                                                `2`="occasional",
                                                `3`="former user",
                                                `4`="never user")

### Quality checks
table(finsote2019_brief$snus_status, finsote2019_brief$smoke_snus_now2,
      useNA = "ifany")
table(finsote2019_brief$ecig_status, finsote2019_brief$smoke_ecig_now,
      useNA = "ifany") #looks good

## Confounders
## Marital status. Convert to factor, create categorical levels
finsote2019_brief$maritalstatus_bin <- as.factor(finsote2019_brief$maritalstatus)
finsote2019_brief$maritalstatus_bin <-  recode_factor(finsote2019_brief$maritalstatus_bin,
                                                      `1`="married or cohabiting",
                                                      `2`="married or cohabiting",
                                                      `3`="separated, single or widowed",
                                                      `4`="separated, single or widowed",
                                                      `5`="separated, single or widowed")

## Mother tongue
finsote2019_brief$mother_tongue <- as.factor(finsote2019_brief$mother_tongue)
finsote2019_brief$mother_tongue <-  recode_factor(finsote2019_brief$mother_tongue,
                                                  `1`="finnish",
                                                  `2`="swedish",
                                                  `3`="other",
                                                  `4`="other")

## Sex.

## Social participation
```

```r
finsote2019_brief$involvement_attend_j <- as.factor(finsote2019_brief$involvement_attend_j)
finsote2019_brief$involvement_attend_j <-  recode_factor(finsote2019_brief$involvement_attend_j,
                                `1`="no participation",
                                `2`="active",
                                `3`="occasional")

# Potential colliders
## BMI
class(finsote2019_brief$height_cm) #with the merge, both variables convert to character
class(finsote2019_brief$weight_kg) #with the merge, both variables convert to character
finsote2019_brief$height_cm <- as.numeric(finsote2019_brief$height_cm)
finsote2019_brief$weight_kg <- as.numeric(finsote2019_brief$weight_kg)
finsote2019_brief <- finsote2019_brief %>%
  mutate(bmi = weight_kg/(height_cm*height_cm/10000))

# Solving data type incompatibilities before appending
finsote1820$involvement_attend_j <- as.factor(finsote1820$involvement_attend_j)

# Creating final baseline dataset
finsote <- bind_rows(finsote1820, finsote2019_brief)
miss_var_summary(finsote) #finally looks good

# Communicable Disease Registry
## Merging
finsote <- left_join(finsote, ttr2, by="GUMM85ID")

## Creating variable date
finsote <- finsote %>%
  mutate(end_date=tilastointipvm) %>%
  mutate(end_date=as.Date(end_date)) %>%
  mutate(tilastointipvm=as.Date(tilastointipvm))%>%
  mutate(end_date=replace(end_date, is.na(tilastointipvm), "2021-09-23"))

## Excludes extreme outliers BMI (see below). This shows when creating a
#dataset with complete cases
finsote <- finsote %>%
  mutate(bmi=replace(bmi, height_cm<137, 1000)) %>%
  mutate(bmi=replace(bmi, height_cm>218, 1000))
finsote$bmi[finsote$bmi==1000]=NA

summary(finsote$height_cm)
## Creates event variable
finsote$Mikrobi
finsote <- finsote %>%
  mutate(event=0) %>%
  mutate(event = replace(event, Mikrobi=="2439-98 Coronavirus", 1)) %>%
  mutate(event = replace(event, Mikrobi=="NA", 1))
```

Exploring missing data

We used several functions of the package *naniar* to explore missing data patterns.

Out of 49186 participants, 10.1% had missing data in any of the variables. The variables with highest frequency of missing data were years of education, smoking status and BMI.

```
# Creates a subset so that miss_var_summary only shows the variables of interest
finsote_missing <- finsote %>%
  dplyr::select(dataid, rg_N_suomi, w_analysis_suomi, rg_stratum_suomi,
                fs_shp_koodi, event, age_cont, sex,
                smoking_status, educ_years, maritalstatus_bin, mother_tongue,
                involvement_attend_j, bmi)
finsote_missing %>%
    group_by(dataid) %>%
  miss_var_summary()


prop_complete(finsote_missing)
pct_complete(finsote_missing)
pct_miss(finsote_missing)
pct_complete_case(finsote_missing)
pct_miss_case(finsote_missing)
miss_case_table(finsote_missing)
```

Exploratory data analysis

We first checked for errors in the data. Some salient issues: - Some people report implausible values of height and weight. One alternative (discussed for example here https://academic.oup.com/ageing/article/39/4/445/8829) is to exclude those above and below 3 SD. An alternative is to use data from a health examination survey that has measured participants using standardized techniques. We opted for the second because, after comparing both alternatives, the second approach was more conservative (i.e. excluded less participants).

Hence, we used data from FINRISK 2012 and converted BMI into missing data in participants with a height below 1.37 mt and higher than 2.18 mt.

```
# Exploratory cross tabs
## Exposures
table(finsote$smoking_status, finsote$dataid)
table(finsote$snus_status, finsote$dataid) #prevalence of snus use is (as we knew) low
table(finsote$ecig_nic_status, finsote$dataid) #very few users
table(finsote$nrt_status, finsote$dataid) #very few users

## Confounders
table(finsote$age_cont, finsote$dataid)
table(finsote$sex, finsote$dataid)
table(finsote$maritalstatus_bin, finsote$dataid)
table(finsote$educ_years, finsote$dataid)
table(finsote$mother_tongue, finsote$dataid)
table(finsote$involvement_attend_j, finsote$dataid)

## Mediators
table(finsote$bmi, finsote$dataid)

#Ridgeplots for continuous variables
## Years of education
```

```r
finsote$dataid <- as.factor(finsote$dataid) #this graphs require a factor variable
ggplot(finsote, aes(x = educ_years, y = dataid, fill = dataid)) +
  geom_density_ridges(scale = 2, rel_min_height = 0.01) +
  labs(title = 'years of education')
ggsave(file="educ_years.png", units="cm", width=25, height=15, dpi=600) #looks good

## Age
ggplot(finsote, aes(x =age_cont, y = dataid, fill = dataid)) +
  geom_density_ridges(scale = 2, rel_min_height = 0.01) +
  labs(title = 'Age')
ggsave(file="age.png", units="cm", width=25, height=15, dpi=600) #looks good

## BMI
ggplot(finsote, aes(x =bmi, y = dataid, fill = dataid)) +
  geom_density_ridges(scale = 2, rel_min_height = 0.01) +
  labs(title = 'Body mass index')
ggsave(file="bmi.png", units="cm", width=25, height=15, dpi=600) #evident problem
#here as some people report some biologically impossible values

#One option is to exclude 3 SD
#quantile(finsote$height_cm, prob=0.997, na.rm=TRUE) #195 cms
#quantile(finsote$height_cm, prob=0.003, na.rm=TRUE) #147 cms
#finsote$height_cm[finsote$height_cm<147]=NA

bmi <- finsote %>%
  dplyr::select(ID, height_cm, weight_kg, bmi)

# Another option is to consider values from a population survey with robust
#measurement methods and exclude based on that. The lowest height in Finrisk 2012
#is 1.372 mts. The tallest person was 2.18 mts
finsote <- finsote %>%
  mutate(bmi=replace(bmi, height_cm<137, 1000)) %>%
  mutate(bmi=replace(bmi, height_cm>218, 1000))
finsote$bmi[finsote$bmi==1000]=NA

## BMI - new graph
ggplot(finsote, aes(x =bmi, y = dataid, fill = dataid)) +
  geom_density_ridges(scale = 2, rel_min_height = 0.01) +
  labs(title = 'Body mass index')
ggsave(file="bmi_new.png", units="cm", width=25, height=15, dpi=600) #better
```

Testing the need for splines

We fitted a minimally adjusted model for sex and the covariate as a linear function or a penalized smoothing spline. We used a likelihood ratio test to compare whether using a spline provides a better fit.

The results show a better fit when using penalised smoothing splines for all variables. We will thus model age, years of education and BMI using splines.

```r
des <- svydesign(id=~1, fpc=~rg_N_suomi, weights=~w_analysis_suomi,
                 strata=~rg_stratum_suomi, data=finsote)
```

```r
# Age
## Age as a linear function
poisson_agelinear <- svyglm(event ~ factor(sex) + age_cont, design=des,
                            family=quasipoisson())
summary(poisson_agelinear)

## Age as a penalised smoothing spline
poisson_agespline <- svyglm(event ~ factor(sex) + pspline(age_cont), design=des,
                            family=quasipoisson())
summary(poisson_agespline)

## Compares coefficients
round(ci.exp(poisson_agelinear), 4)
round(ci.exp(poisson_agespline), 4)

## Likelihood ratio test
anova(poisson_agelinear, poisson_agespline, method="Wald") #p=< 2.22e-16, better fit as a spline

# Years of education
poisson_edulinear <- svyglm(event ~ factor(sex) + pspline(age_cont) + educ_years,
                           design=des, family=quasipoisson())
summary(poisson_edulinear)

poisson_eduspline <- svyglm(event ~ factor(sex) + pspline(age_cont) +
                              pspline(educ_years),
                      design=des, family=quasipoisson())
summary(poisson_eduspline)

round(ci.exp(poisson_edulinear), 4)
round(ci.exp(poisson_eduspline), 4)

anova(poisson_edulinear, poisson_eduspline, method="Wald") #p=< 2.22e-16, better fit than linear functi


# BMI
poisson_bmilinear <- svyglm(event ~ factor(sex) + pspline(age_cont)+ bmi,
                            design=des, family=quasipoisson())
poisson_bmilinear

poisson_bmispline <- svyglm(event ~ factor(sex) + pspline(age_cont) +
                              pspline(bmi),
                            design=des, family=quasipoisson())
poisson_bmispline

round(ci.exp(poisson_bmilinear), 4)
round(ci.exp(poisson_bmispline), 4)

anova(poisson_bmilinear, poisson_bmispline, method="Wald") #p=< 2.22e-16,
#better fit than linear function
```

Main analysis

Creates a subset of complete cases and incorporates survey design

The following section restricts the data to complete cases for all covariates and weights variables. The models automatically exclude those with missing data on the outcome.

This overall dataset has 45391 participants, but the final analytical sample for each model depends on the missingness of the outcome too.

```
# Creates subset of complete cases
finsotecc <- finsote %>%
  dplyr::select(rg_N_suomi, w_analysis_suomi, rg_stratum_suomi, dataid, fs_shp_koodi,
                event, age_cont, sex, smoking_status, snus_status, ecig_nic_status,
                ecig_nonic_status, nrt_status,
                educ_years, maritalstatus_bin, mother_tongue,
                involvement_attend_j, bmi, end_date) %>%
  filter(complete.cases(rg_N_suomi, w_analysis_suomi, rg_stratum_suomi, fs_shp_koodi,
                event, age_cont, sex, educ_years, maritalstatus_bin, mother_tongue,
                involvement_attend_j, bmi))

# Combines daily and occasional users
table(finsotecc$snus_status, useNA = "ifany")
finsotecc$snus_status <- recode_factor(finsotecc$snus_status,
                                       "daily user" = "current user")
finsotecc$snus_status <- recode_factor(finsotecc$snus_status,
                                       "occasional" = "current user")

finsotecc$ecig_nic_status <- recode_factor(finsotecc$ecig_nic_status,
                                           "daily user" = "current user")
finsotecc$ecig_nic_status <- recode_factor(finsotecc$ecig_nic_status,
                                           "occasional" = "current user")
finsotecc$ecig_nonic_status <- recode_factor(finsotecc$ecig_nonic_status,
                                             "daily user" = "current user")
finsotecc$ecig_nonic_status <- recode_factor(finsotecc$ecig_nonic_status,
                                             "occasional" = "current user")
finsotecc$nrt_status <- recode_factor(finsotecc$nrt_status,
                                      "daily user" = "current user")
finsotecc$nrt_status <- recode_factor(finsotecc$nrt_status,
                                      "occasional" = "current user")

# Reference levels
finsotecc <- within(finsotecc, smoking_status <- relevel(smoking_status,
                                                         ref = 'never smoker'))
finsotecc <- within(finsotecc, snus_status <- relevel(snus_status,
                                                      ref = 'never user'))
finsotecc <- within(finsotecc, ecig_nic_status <- relevel(ecig_nic_status,
                                                          ref = 'never user'))
finsotecc <- within(finsotecc, ecig_nonic_status <- relevel(ecig_nonic_status,
                                                            ref = 'never user'))
finsotecc <- within(finsotecc, nrt_status <- relevel(nrt_status,
                                                     ref = 'never user'))

# Setting up survey design (weights, strata and FPC)
```

```
des_final <- svydesign(id=~1, fpc=~rg_N_suomi, weights=~w_analysis_suomi,
                       strata=~rg_stratum_suomi, data=finsotecc)
```

Table 1

We used the function *svyCreateTableOne* from the package *tableone* to produce cross-tabulations that take the complex survey design into account

```
# Table 1
table(finsotecc$event, finsotecc$smoking_status)
table(finsotecc$smoking_status)
table(finsotecc$event)

listVars <- c("event", "sex", "age_cont", "maritalstatus_bin", "educ_years",
              "mother_tongue", "involvement_attend_j", "bmi")
catVars <- c("event", "sex", "maritalstatus_bin", "mother_tongue")
table1 <- svyCreateTableOne(listVars, strata=c("smoking_status"), factor=catVars,
                            test=FALSE, data=des_final)
tab1 <- print(table1, printToggle=FALSE, nospaces=TRUE, format="p", contDigits=1)
kable(tab1, format.args=list(digits=1, nsmall=1, format))

table(finsotecc$smoking_status)
# Table S2 for snus
table(finsotecc$event, finsotecc$snus_status)
table(finsotecc$snus_status)

listVars <- c("sex", "age_cont", "maritalstatus_bin", "educ_years", "mother_tongue",
              "involvement_attend_j", "bmi")
catVars <- c("sex", "maritalstatus_bin", "mother_tongue")
table1 <- svyCreateTableOne(listVars, strata=c("snus_status"), factor=catVars,
                            test=FALSE, data=des_final)
tab1 <- print(table1, printToggle=FALSE, nospaces=TRUE, format="p", contDigits=1)
kable(tab1, format.args=list(digits=4, nsmall=2, format))

# Table S3 for e-cigarettes with nicotine
table(finsotecc$event, finsotecc$ecig_nic_status)
table(finsotecc$ecig_nic_status)

listVars <- c("sex", "age_cont", "maritalstatus_bin", "educ_years", "mother_tongue",
              "involvement_attend_j", "bmi")
catVars <- c("sex", "maritalstatus_bin", "mother_tongue")
table1 <- svyCreateTableOne(listVars, strata=c("ecig_nic_status"), factor=catVars,
                            test=FALSE, data=des_final)
tab1 <- print(table1, printToggle=FALSE, nospaces=TRUE, format="p", contDigits=1)
kable(tab1, format.args=list(digits=4, nsmall=2, format))

# Table S4 for e-cigarettes without nicotine
table(finsotecc$event, finsotecc$ecig_nonic_status)
table(finsotecc$ecig_nonic_status)

listVars <- c("sex", "age_cont", "maritalstatus_bin", "educ_years", "mother_tongue",
              "involvement_attend_j", "bmi")
```

```r
catVars <- c("sex", "maritalstatus_bin", "mother_tongue")
table1 <- svyCreateTableOne(listVars, strata=c("ecig_nonic_status"), factor=catVars,
                            test=FALSE, data=des_final)
tab1 <- print(table1, printToggle=FALSE, nospaces=TRUE, format="p", contDigits=1)
kable(tab1, format.args=list(digits=4, nsmall=2, format))

# Table S5 for nicotine replacement therapy
table(finsotecc$event, finsotecc$nrt_status)
table(finsotecc$nrt_status)

listVars <- c("sex", "age_cont", "maritalstatus_bin", "educ_years", "mother_tongue",
              "involvement_attend_j", "bmi")
catVars <- c("sex", "maritalstatus_bin", "mother_tongue")
table1 <- svyCreateTableOne(listVars, strata=c("nrt_status"), factor=catVars,
                            test=FALSE, data=des_final)
tab1 <- print(table1, printToggle=FALSE, nospaces=TRUE, format="p", contDigits=1)
kable(tab1, format.args=list(digits=4, nsmall=2, format))
```

Main analysis

Smoking status

```r
# Smoking
## Create a subset where participants with missing data on smoking
#(smoking_status==NA) are excluded
finsotecc <- within(finsotecc, smoking_status <- relevel(smoking_status,
                                                          ref = 'never smoker'))
des_final <- svydesign(id=~1, fpc=~rg_N_suomi, weights=~w_analysis_suomi,
                       strata=~rg_stratum_suomi, data=finsotecc)

## Smoking first column
table(finsotecc$smoking_status, useNA = "ifany")
table(finsotecc$smoking_status, finsotecc$event, useNA = "ifany")

## Crude model
m1_poisson <- svyglm(event ~ smoking_status, design=des_final,
                     family=quasipoisson())
m1_poisson <- svyglm(event ~ factor(smoking_status), design=des_final,
                     family=quasipoisson())
summary(m1_poisson)

## Adjusted for age and sex
m2_poisson <- svyglm(event ~ factor(smoking_status) + factor(sex) +
                       pspline(age_cont),
                       design=des_final, family=quasipoisson())
summary(m2_poisson)

## Adjusted for confounders
m3_poisson <- svyglm(event ~ factor(smoking_status) + factor(sex) +
                       pspline(age_cont) +
```

```
                        pspline(educ_years) + factor(maritalstatus_bin) +
                        factor(mother_tongue) + factor(involvement_attend_j) +
                        factor(fs_shp_koodi),
                        design=des_final, family=quasipoisson())
summary(m3_poisson)

## Adjusted for potential collider
m4_poisson <- svyglm(event ~ factor(smoking_status) + factor(sex) +
                        pspline(age_cont) +
                        pspline(educ_years) + factor(maritalstatus_bin) +
                        factor(mother_tongue) + factor(involvement_attend_j) +
                        factor(fs_shp_koodi) +
                        pspline(bmi),
                        design=des_final, family=quasipoisson())
summary(m4_poisson)

#Obtains main estimates
kable(round(ci.exp(m1_poisson), 2))
kable(round(ci.exp(m2_poisson), 2))
kable(round(ci.exp(m3_poisson), 2))
kable(round(ci.exp(m4_poisson), 2))

n1_smok <- m1_poisson$model[1]
n2_smok <- m2_poisson$model[1]
n3_smok <- m3_poisson$model[1]
n4_smok <- m4_poisson$model[1] #looks good, n=44199
```

Snus (smokeless tobacco)

```
# Snus first column
table(finsotecc$snus_status, useNA = "ifany")
table(finsotecc$snus_status, finsotecc$event, useNA = "ifany")

## Crude model
m1_poisson_snus <- svyglm(event ~ factor(snus_status),
                           design=des_final, family=quasipoisson())
summary(m1_poisson_snus)

## Adjusted for age and sex
m2_poisson_snus <- svyglm(event ~ factor(snus_status) + factor(sex) +
                             pspline(age_cont),
                           design=des_final, family=quasipoisson())
summary(m2_poisson_snus)

## Adjusted for confounders
m3_poisson_snus <- svyglm(event ~ factor(snus_status) + factor(sex) +
                        pspline(age_cont) +
                        pspline(educ_years) + factor(maritalstatus_bin) +
                        factor(mother_tongue) + factor(involvement_attend_j) +
                        factor(fs_shp_koodi),
                        design=des_final, family=quasipoisson())
```

```r
summary(m3_poisson_snus)

## Adjusted for potential colliders
m4_poisson_snus <- svyglm(event ~ factor(snus_status) + factor(sex) +
                       pspline(age_cont) +
                       pspline(educ_years) + factor(maritalstatus_bin) +
                       factor(mother_tongue) + factor(involvement_attend_j) +
                       factor(fs_shp_koodi) + pspline(bmi),
                       design=des_final, family=quasipoisson())
summary(m4_poisson_snus)

#Obtains main estimates
kable(round(ci.exp(m1_poisson_snus), 2))
kable(round(ci.exp(m2_poisson_snus), 2))
kable(round(ci.exp(m3_poisson_snus), 2))
kable(round(ci.exp(m4_poisson_snus), 2))

# Checking that estimates are on the same sample
n1_snus <- m1_poisson_snus$model[1]
n2_snus <- m2_poisson_snus$model[1]
n3_snus <- m3_poisson_snus$model[1]
n4_snus <- m4_poisson_snus$model[1] #looks good, n=32931
```

E-cigarettes with nicotine

```r
# E-cigarettes with nicotine
table(finsotecc$ecig_nic_status, useNA = "ifany")
table(finsotecc$ecig_nic_status, finsotecc$event, useNA = "ifany")

## Crude model
m1_poisson_ecig_nic <- svyglm(event ~ factor(ecig_nic_status), design=des_final,
                              family=quasipoisson())
summary(m1_poisson_ecig_nic)

## Adjusted for age and sex
m2_poisson_ecig_nic <- svyglm(event ~ factor(ecig_nic_status) + factor(sex) +
                                pspline(age_cont), design=des_final,
                              family=quasipoisson())
summary(m2_poisson_ecig_nic)

## Adjusted for confounders
m3_poisson_ecig_nic <- svyglm(event ~ factor(ecig_nic_status) + factor(sex) +
                       pspline(age_cont) +
                       pspline(educ_years) + factor(maritalstatus_bin) +
                       factor(mother_tongue) + factor(involvement_attend_j) +
                       factor(fs_shp_koodi),
                       design=des_final, family=quasipoisson())
summary(m3_poisson_ecig_nic)

## Adjusted for potential colliders
m4_poisson_ecig_nic <- svyglm(event ~ factor(ecig_nic_status) + factor(sex) +
```

```
                        pspline(age_cont) +
                        pspline(educ_years) + factor(maritalstatus_bin) +
                        factor(mother_tongue) + factor(involvement_attend_j) +
                        factor(fs_shp_koodi) +
                        pspline(bmi),
                        design=des_final, family=quasipoisson())
summary(m4_poisson_ecig_nic)

#Obtains main estimates
kable(round(ci.exp(m1_poisson_ecig_nic), 2))
kable(round(ci.exp(m2_poisson_ecig_nic), 2))
kable(round(ci.exp(m3_poisson_ecig_nic), 2))
kable(round(ci.exp(m4_poisson_ecig_nic), 2))

# Checking that estimates are on the same sample
n1_ecig_nic <- m1_poisson_ecig_nic$model[1]
n2_ecig_nic <- m2_poisson_ecig_nic$model[1]
n3_ecig_nic <- m3_poisson_ecig_nic$model[1]
n4_ecig_nic <- m4_poisson_ecig_nic$model[1] #looks good, n=27098
```

E-cigarettes without nicotine

```
# E-cigarettes without nicotine
table(finsotecc$ecig_nonic_status, useNA = "ifany")
table(finsotecc$ecig_nonic_status, finsotecc$event, useNA = "ifany")

## Crude model
m1_poisson_ecig_no_nic <- svyglm(event ~ factor(ecig_nonic_status),
                                 design=des_final,
                                 family=quasipoisson())
summary(m1_poisson_ecig_no_nic)

## Adjusted for age and sex
m2_poisson_ecig_no_nic <- svyglm(event ~ factor(ecig_nonic_status) + factor(sex) +
                                   pspline(age_cont), design=des_final,
                                 family=quasipoisson())
summary(m2_poisson_ecig_no_nic)

## Adjusted for confounders
m3_poisson_ecig_no_nic <- svyglm(event ~ factor(ecig_nonic_status) + factor(sex) +
                        pspline(age_cont) +
                        pspline(educ_years) + factor(maritalstatus_bin) +
                        factor(mother_tongue) + factor(involvement_attend_j) +
                        factor(fs_shp_koodi),
                        design=des_final, family=quasipoisson())
summary(m3_poisson_ecig_no_nic)

## Adjusted for potential colliders
m4_poisson_ecig_no_nic <- svyglm(event ~ factor(ecig_nonic_status) + factor(sex) +
                        pspline(age_cont) +
                        pspline(educ_years) + factor(maritalstatus_bin) +
```

```
                         factor(mother_tongue) + factor(involvement_attend_j) +
                         factor(fs_shp_koodi) +
                         pspline(bmi), design=des_final, family=quasipoisson())
summary(m4_poisson_ecig_no_nic)

#Obtains main estimates
kable(round(ci.exp(m1_poisson_ecig_no_nic), 2))
kable(round(ci.exp(m2_poisson_ecig_no_nic), 2))
kable(round(ci.exp(m3_poisson_ecig_no_nic), 2))
kable(round(ci.exp(m4_poisson_ecig_no_nic), 2))

# Checking that estimates are on the same sample
n1_ecig_nonic <- m1_poisson_ecig_no_nic$model[1]
n2_ecig_nonic <- m2_poisson_ecig_no_nic$model[1]
n3_ecig_nonic <- m3_poisson_ecig_no_nic$model[1]
n4_ecig_nonic <- m4_poisson_ecig_no_nic$model[1] #looks good, n=27005
```

Nicotine replacement therapy products

```
# NRT first column
table(finsotecc$nrt_status, useNA = "ifany")
table(finsotecc$nrt_status, finsotecc$event, useNA = "ifany")

## Crude model
m1_poisson_nrt <- svyglm(event ~ factor(nrt_status), design=des_final,
                           family=quasipoisson())
summary(m1_poisson_nrt)

## Adjusted for age and sex
m2_poisson_nrt <- svyglm(event ~ factor(nrt_status) + factor(sex) +
                             pspline(age_cont),
                           design=des_final, family=quasipoisson())
summary(m2_poisson_nrt)

## Adjusted for confounders
m3_poisson_nrt <- svyglm(event ~ factor(nrt_status) + factor(sex) +
                         pspline(age_cont) +
                         pspline(educ_years) + factor(maritalstatus_bin) +
                         factor(mother_tongue) + factor(involvement_attend_j) +
                         factor(fs_shp_koodi),
                         design=des_final, family=quasipoisson())
summary(m3_poisson_nrt)

## Adjusted for potential colliders
m4_poisson_nrt <- svyglm(event ~ factor(nrt_status) + factor(sex) +
                         pspline(age_cont) +
                         pspline(educ_years) + factor(maritalstatus_bin) +
                         factor(mother_tongue) + factor(involvement_attend_j) +
                         factor(fs_shp_koodi) +
                         pspline(bmi), design=des_final, family=quasipoisson())
summary(m4_poisson_nrt)
```

```
#Obtains main estimates
kable(round(ci.exp(m1_poisson_nrt), 2))
kable(round(ci.exp(m2_poisson_nrt), 2))
kable(round(ci.exp(m3_poisson_nrt), 2))
kable(round(ci.exp(m4_poisson_nrt), 2))

# Checking that estimates are on the same sample
n1_nrt <- m1_poisson_nrt$model[1]
n2_nrt <- m2_poisson_nrt$model[1]
n3_nrt <- m3_poisson_nrt$model[1]
n4_nrt <- m4_poisson_nrt$model[1] #looks good, n=27073
```

Sensitivity analysis

```
finsote1819cc <- finsotecc %>%
  filter(dataid==2018 | dataid==2019) %>%
  mutate(start_date2="2020-02-26")
finsote1819cc$start_date <- as.Date(finsote1819cc$start_date2)

table(finsote1819cc$dataid)
table(finsotecc$dataid)

# Combines daily and occasional users
table(finsote1819cc$snus_status, useNA = "ifany")
finsote1819cc$snus_status <- recode_factor(finsote1819cc$snus_status,
                                           "daily user" = "current user")
finsote1819cc$snus_status <- recode_factor(finsote1819cc$snus_status,
                                           "occasional" = "current user")

finsote1819cc$ecig_nic_status <- recode_factor(finsote1819cc$ecig_nic_status,
                                               "daily user" = "current user")
finsote1819cc$ecig_nic_status <- recode_factor(finsote1819cc$ecig_nic_status,
                                               "occasional" = "current user")
finsote1819cc$ecig_nonic_status <- recode_factor(finsote1819cc$ecig_nonic_status,
                                                 "daily user" = "current user")
finsote1819cc$ecig_nonic_status <- recode_factor(finsote1819cc$ecig_nonic_status,
                                                 "occasional" = "current user")
finsote1819cc$nrt_status <- recode_factor(finsote1819cc$nrt_status,
                                          "daily user" = "current user")
finsote1819cc$nrt_status <- recode_factor(finsote1819cc$nrt_status,
                                          "occasional" = "current user")

# Reference levels
finsote1819cc <- within(finsote1819cc, smoking_status <- relevel(smoking_status,
                                                                 ref = 'never smoker'))
finsote1819cc <- within(finsote1819cc, snus_status <- relevel(snus_status,
                                                              ref = 'never user'))
finsote1819cc <- within(finsote1819cc, ecig_nic_status <- relevel(ecig_nic_status,
                                                                  ref = 'never user'))
finsote1819cc <- within(finsote1819cc, ecig_nonic_status <- relevel(ecig_nonic_status,
```

```
                                                            ref = 'never user'))
finsote1819cc <- within(finsote1819cc, nrt_status <- relevel(nrt_status,
                                                            ref = 'never user'))

# Setting up survey design (weights, strata and FPC)

des1819_final <- svydesign(id=~1, fpc=~rg_N_suomi, weights=~w_analysis_suomi,
                           strata=~rg_stratum_suomi, data=finsote1819cc)
```

Sensitivity analysis (i): restricts analysis to prospectively collected data to examine information bias

This sensitivity analysis replicates the main analysis but restricting the data to FinSote 2018 and 2019. All models run, but some report convergence problems when including BMI.

Smoking

```
# Smoking
des1819_final <- svydesign(id=~1, fpc=~rg_N_suomi, weights=~w_analysis_suomi,
                           strata=~rg_stratum_suomi, data=finsote1819cc)

## Smoking first column
table(finsote1819cc$smoking_status, useNA = "ifany")
table(finsote1819cc$smoking_status, finsote1819cc$event, useNA = "ifany")

## Creates list of variables
smok <- "factor(smoking_status)"
snus <- "factor(snus_status)"
ecig_nic <- "factor(ecig_nic_status)"
ecig_nonic <- "factor(ecig_nonic_status)"
nrt <- "factor(nrt_status)"
m2_variables <- c("factor(sex)", "pspline(age_cont)")
m3_variables <- c("factor(sex)", "pspline(age_cont)", "pspline(educ_years)",
                  "factor(maritalstatus_bin)", "factor(mother_tongue)",
                  "factor(involvement_attend_j)", "factor(fs_shp_koodi)")
m4_variables <- c("factor(sex)", "pspline(age_cont)", "pspline(educ_years)",
                  "factor(maritalstatus_bin)", "factor(mother_tongue)",
                  "factor(involvement_attend_j)", "factor(fs_shp_koodi)",
                  "pspline(bmi)")

## Run models
m1_smok1819 <- svyglm(as.formula(paste("event ~", paste(smok, collapse="+"))),
                                      design=des1819_final, family=quasipoisson())
summary(m1_smok1819)

m2_smok1819 <- svyglm(as.formula(paste("event ~", paste(smok, m2_variables, sep="+",
                                                        collapse="+"))),
                                      design=des1819_final, family=quasipoisson())
summary(m2_smok1819)
```

```r
m3_smok1819 <- svyglm(as.formula(paste("event ~", paste(smok, m3_variables, sep="+",
                                                          collapse="+"))),
                                        design=des1819_final, family=quasipoisson())
summary(m3_smok1819)

m4_smok1819 <- svyglm(as.formula(paste("event ~", paste(smok, m4_variables, sep="+",
                                                          collapse="+"))),
                                        design=des1819_final, family=quasipoisson())
summary(m4_smok1819)

#Obtains main estimates
kable(round(ci.exp(m1_smok1819), 2))
kable(round(ci.exp(m2_smok1819), 2))
kable(round(ci.exp(m3_smok1819), 2))
kable(round(ci.exp(m4_smok1819), 2))

n1_smok1819 <- m1_smok1819$model[1]
n2_smok1819 <- m2_smok1819$model[1]
n3_smok1819 <- m3_smok1819$model[1]
n4_smok1819 <- m4_smok1819$model[1] #n=19102
```

Snus

```r
# Snus use
## snus first column
table(finsote1819cc$snus_status, useNA = "ifany")
table(finsote1819cc$snus_status, finsote1819cc$event, useNA = "ifany")

## Run models
m1_snus1819 <- svyglm(as.formula(paste("event ~", paste(snus, collapse="+"))),
                                        design=des1819_final, family=quasipoisson())
summary(m1_snus1819)
m2_snus1819 <- svyglm(as.formula(paste("event ~", paste(snus, m2_variables, sep="+",
                                                          collapse="+"))),
                                        design=des1819_final, family=quasipoisson())
summary(m2_snus1819)
m3_snus1819 <- svyglm(as.formula(paste("event ~", paste(snus, m3_variables, sep="+",
                                                          collapse="+"))),
                                        design=des1819_final, family=quasipoisson())
summary(m3_snus1819)
m4_snus1819 <- svyglm(as.formula(paste("event ~", paste(snus, m4_variables, sep="+",
                                                          collapse="+"))),
                                        design=des1819_final, family=quasipoisson())
summary(m4_snus1819)

## Obtains main estimates
kable(round(ci.exp(m1_snus1819), 2))
kable(round(ci.exp(m2_snus1819), 2))
kable(round(ci.exp(m3_snus1819), 2))
kable(round(ci.exp(m4_snus1819), 2))

n1_snus1819 <- m1_snus1819$model[1]
```

```
n2_snus1819 <- m2_snus1819$model[1]
n3_snus1819 <- m3_snus1819$model[1]
n4_snus1819 <- m4_snus1819$model[1] #n=15974
```

E-cigarettes with nicotine

```
# E-cigarettes with nicotine

## First column
table(finsote1819cc$ecig_nic_status, useNA = "ifany")
table(finsote1819cc$ecig_nic_status, finsote1819cc$event, useNA = "ifany")

## Run models
m1_ecig_nic1819 <- svyglm(as.formula(paste("event ~", paste(ecig_nic, collapse="+"))),
                                       design=des1819_final, family=quasipoisson())
summary(m1_ecig_nic1819)
m2_ecig_nic1819 <- svyglm(as.formula(paste("event ~", paste(ecig_nic, m2_variables,
                                                      sep="+", collapse="+"))),
                                       design=des1819_final, family=quasipoisson())
summary(m2_ecig_nic1819)
m3_ecig_nic1819 <- svyglm(as.formula(paste("event ~", paste(ecig_nic, m3_variables,
                                                      sep="+", collapse="+"))),
                                       design=des1819_final, family=quasipoisson())
summary(m3_ecig_nic1819)
m4_ecig_nic1819 <- svyglm(as.formula(paste("event ~", paste(ecig_nic, m4_variables,
                                                      sep="+", collapse="+"))),
                                       design=des1819_final, family=quasipoisson())
summary(m4_ecig_nic1819)

## Obtains main estimates
kable(round(ci.exp(m1_ecig_nic1819), 2))
kable(round(ci.exp(m2_ecig_nic1819), 2))
kable(round(ci.exp(m3_ecig_nic1819), 2))
kable(round(ci.exp(m4_ecig_nic1819), 2))

n1_ecig_nic1819 <- m1_ecig_nic1819$model[1]
n2_ecig_nic1819 <- m2_ecig_nic1819$model[1]
n3_ecig_nic1819 <- m3_ecig_nic1819$model[1]
n4_ecig_nic1819 <- m4_ecig_nic1819$model[1] #n=10181
```

E-cigarettes without nicotine

```
# E-cigarettes without nicotine

## First column
table(finsote1819cc$ecig_nonic_status, useNA = "ifany")
table(finsote1819cc$ecig_nonic_status, finsote1819cc$event, useNA = "ifany")

## Run models
m1_ecig_nonic1819 <- svyglm(as.formula(paste("event ~", paste(ecig_nonic, collapse="+"))),
```

```
                                    design=des1819_final, family=quasipoisson())
summary(m1_ecig_nonic1819)

m2_ecig_nonic1819 <- svyglm(as.formula(paste("event ~", paste(ecig_nonic, m2_variables,
                                                         sep="+", collapse="+"))),
                                    design=des1819_final, family=quasipoisson())
summary(m2_ecig_nonic1819)

m3_ecig_nonic1819 <- svyglm(as.formula(paste("event ~", paste(ecig_nonic, m3_variables,
                                                         sep="+", collapse="+"))),
                                    design=des1819_final, family=quasipoisson())
summary(m3_ecig_nonic1819)

m4_ecig_nonic1819 <- svyglm(as.formula(paste("event ~", paste(ecig_nonic, m4_variables,
                                                         sep="+", collapse="+"))),
                                    design=des1819_final, family=quasipoisson())
summary(m4_ecig_nonic1819)

## Obtains main estimates
kable(round(ci.exp(m1_ecig_nonic1819), 2))
kable(round(ci.exp(m2_ecig_nonic1819), 2))
kable(round(ci.exp(m3_ecig_nonic1819), 2))
kable(round(ci.exp(m4_ecig_nonic1819), 2))

n1_ecig_nonic1819 <- m1_ecig_nonic1819$model[1]
n2_ecig_nonic1819 <- m2_ecig_nonic1819$model[1]
n3_ecig_nonic1819 <- m3_ecig_nonic1819$model[1]
n4_ecig_nonic1819 <- m4_ecig_nonic1819$model[1] #n=10155
```

Nicotine replacement therapy products

```
# Nicotine replacement products

table(finsote1819cc$nrt_status, useNA = "ifany")
table(finsote1819cc$nrt_status, finsote1819cc$event, useNA = "ifany")

## Run models
m1_nrt1819 <- svyglm(as.formula(paste("event ~", paste(nrt, collapse="+"))),
                                    design=des1819_final, family=quasipoisson())
summary(m1_nrt1819)
m2_nrt1819 <- svyglm(as.formula(paste("event ~", paste(nrt, m2_variables, sep="+",
                                                collapse="+"))),
                                    design=des1819_final, family=quasipoisson())
summary(m2_nrt1819)
m3_nrt1819 <- svyglm(as.formula(paste("event ~", paste(nrt, m3_variables, sep="+",
                                                collapse="+"))),
                                    design=des1819_final, family=quasipoisson())
summary(m3_nrt1819)
m4_nrt1819 <- svyglm(as.formula(paste("event ~", paste(nrt, m4_variables, sep="+",
                                                collapse="+"))),
                                    design=des1819_final, family=quasipoisson())
summary(m4_nrt1819)
```

```
## Obtains main estimates
kable(round(ci.exp(m1_nrt1819), 2))
kable(round(ci.exp(m2_nrt1819), 2))
kable(round(ci.exp(m3_nrt1819), 2))
kable(round(ci.exp(m4_nrt1819), 2))

n1_nrt1819 <- m1_nrt1819$model[1]
n2_nrt1819 <- m2_nrt1819$model[1]
n3_nrt1819 <- m3_nrt1819$model[1]
n4_nrt1819 <- m4_nrt1819$model[1] #n=10187
```

Sensitivity analysis (ii): same analyses restricted to prospectively collected data but using Cox proportional hazards models to follow pre-registered analyses

This sensitivity analysis replicates the main analysis but restricting the data to FinSote 2018 and 2019 and using Cox proportional hazards models. Pre-registration can be found in ClinicalTrials.gov (NCT04915781).

Exploratory survival analysis

```
# Setting up time variable
finsote1819cc <- finsote1819cc %>%
  mutate(time=as.numeric(difftime(end_date, start_date2, units="days")))

#Events and exploratory survival analysis
table(finsote1819cc$event, finsote1819cc$smoking_status)
table(finsote1819cc$event, finsote1819cc$dataid)
finsote1819cc %>%
    tabyl(smoking_status, event, dataid) %>%
  kable(digits = 1, caption = "Events by smoking status and survey wave",
        format = "html")

survRate(Surv(time, event)~1, data=finsote1819cc)
survRate(Surv(time, event)~smoking_status, data=finsote1819cc)
survRate(Surv(time, event)~snus_status, data=finsote1819cc)
survRate(Surv(time, event)~ecig_nic_status, data=finsote1819cc)
survRate(Surv(time, event)~ecig_nonic_status, data=finsote1819cc)
survRate(Surv(time, event)~nrt_status, data=finsote1819cc)

KM_fit <- survfit(Surv(time, event) ~ 1, data = finsote1819cc)
KM_fit
autoplot(KM_fit)

KM_smoke_fit <- survfit(Surv(time, event) ~ smoking_status, data = finsote1819cc)
autoplot(KM_smoke_fit)

# Log rank tests
logrank <- survdiff(Surv(time, event) ~ smoking_status, data = finsote1819cc)
logrank
```

Cox proportional hazard models

We ran Cox proportional hazard models using the same models and data as in sensitivity analysis (ii). Models 3 and 4 run into singular fit problems and thus, to obtain a coefficient, we modelled the variables as linear predictors instead of using penalised smoothing splines. In the case of the models with e-cigarettes with and without nicotine and nicotine replacement therapy, we also had to remove the fixed effects for hospital districts to run the models.

We tested the proportional hazards assumption by plotting Schoenfeld residuals against time. Using R version 3.6.3 the results showed that the continuous variables mildly violate the proportional hazards function, as their effect seems to slightly decrease with time. This indicates, again, that the models did not fit well, as there are very few events in the data. The obtained coefficients, anyhow, seem to be reasonably similar to the ones obtained using a Poisson regression.

```
des1819_final <- svydesign(id=~1, fpc=~rg_N_suomi, weights=~w_analysis_suomi,
                           strata=~rg_stratum_suomi, data=finsote1819cc)

## Creates list of variables
smok <- "factor(smoking_status)"
snus <- "factor(snus_status)"
ecig_nic <- "factor(ecig_nic_status)"
ecig_nonic <- "factor(ecig_nonic_status)"
nrt <- "factor(nrt_status)"
m2_variables_cox <- c("factor(sex)", "pspline(age_cont)")
m3_variables_cox <- c("factor(sex)", "age_cont", "educ_years",
                  "factor(maritalstatus_bin)", "factor(mother_tongue)",
                  "factor(involvement_attend_j)", "factor(fs_shp_koodi)")
m4_variables_cox <- c("factor(sex)", "age_cont", "educ_years",
                  "factor(maritalstatus_bin)", "factor(mother_tongue)",
                  "factor(involvement_attend_j)", "factor(fs_shp_koodi)", "bmi")

# Smoking status
## Runs models
m1_smok1819_cox <- svycoxph(as.formula(paste("Surv(time, event) ~", paste(smok,
                                       collapse="+"))),
                                       design=des1819_final)
summary(m1_smok1819_cox)

m2_smok1819_cox <- svycoxph(as.formula(paste("Surv(time, event) ~", paste(smok,
                    m2_variables_cox, sep="+", collapse="+"))),
                                       design=des1819_final)
summary(m2_smok1819_cox)

m3_smok1819_cox <- svycoxph(as.formula(paste("Surv(time, event) ~", paste(smok,
                    m3_variables_cox, sep="+", collapse="+"))),
                                       design=des1819_final)
summary(m3_smok1819_cox)

m4_smok1819_cox <- svycoxph(as.formula(paste("Surv(time, event) ~", paste(smok,
                    m4_variables_cox, sep="+", collapse="+"))),
                                       design=des1819_final)
summary(m4_smok1819_cox)

## Obtains main estimates
```

```
kable(round(ci.exp(m1_smok1819_cox), 2))
kable(round(ci.exp(m2_smok1819_cox), 2))
kable(round(ci.exp(m3_smok1819_cox), 2))
kable(round(ci.exp(m4_smok1819_cox), 2))

n1_smok1819_cox <- m1_smok1819_cox$model[1]
n2_smok1819_cox <- m2_smok1819_cox$model[1]
n3_smok1819_cox <- m3_smok1819_cox$model[1]
n4_smok1819_cox <- m4_smok1819_cox$model[1] #n=19102

# Snus status
## Runs models
m1_snus1819_cox <- svycoxph(as.formula(paste("Surv(time, event) ~", paste(snus,
                                      collapse="+"))),
                                       design=des1819_final)
summary(m1_snus1819_cox)
m2_snus1819_cox <- svycoxph(as.formula(paste("Surv(time, event) ~", paste(snus,
                    m2_variables_cox, sep="+", collapse="+"))),
                                       design=des1819_final)
summary(m2_snus1819_cox)
m3_snus1819_cox <- svycoxph(as.formula(paste("Surv(time, event) ~", paste(snus,
                    m3_variables_cox, sep="+", collapse="+"))),
                                       design=des1819_final)
summary(m3_snus1819_cox)
m4_snus1819_cox <- svycoxph(as.formula(paste("Surv(time, event) ~", paste(snus,
                    m4_variables_cox, sep="+", collapse="+"))),
                                       design=des1819_final)
summary(m4_snus1819_cox)

## Obtains main estimates
kable(round(ci.exp(m1_snus1819_cox), 2))
kable(round(ci.exp(m2_snus1819_cox), 2))
kable(round(ci.exp(m3_snus1819_cox), 2))
kable(round(ci.exp(m4_snus1819_cox), 2))

n1_snus1819_cox <- m1_snus1819_cox$model[1]
n2_snus1819_cox <- m2_snus1819_cox$model[1]
n3_snus1819_cox <- m3_snus1819_cox$model[1]
n4_snus1819_cox <- m4_snus1819_cox$model[1] #n=15974

# E-cig with nicotine status
## Runs models
m1_ecig_nic1819_cox <- svycoxph(as.formula(paste("Surv(time, event) ~",
                                                 paste(ecig_nic, collapse="+"))),
                                       design=des1819_final)
summary(m1_ecig_nic1819_cox)
m2_ecig_nic1819_cox <- svycoxph(as.formula(paste("Surv(time, event) ~",
                                                 paste(ecig_nic, m2_variables_cox,
                                                       sep="+", collapse="+"))),
                                       design=des1819_final)
summary(m2_ecig_nic1819_cox)

#Alternative m3 formulation
```

```r
m3_variables_simpl <- c("factor(sex)", "age_cont", "educ_years",
                  "factor(maritalstatus_bin)", "factor(mother_tongue)",
                  "factor(involvement_attend_j)")
m4_variables_simpl <- c("factor(sex)", "age_cont", "educ_years",
                  "factor(maritalstatus_bin)", "factor(mother_tongue)",
                  "factor(involvement_attend_j)", "bmi")
m3_ecig_nic1819_cox <- svycoxph(as.formula(paste("Surv(time, event) ~",
                                                 paste(ecig_nic, m3_variables_simpl,
                                                       sep="+", collapse="+"))),
                                          design=des1819_final)
summary(m3_ecig_nic1819_cox)
m4_ecig_nic1819_cox <- svycoxph(as.formula(paste("Surv(time, event) ~",
                                                 paste(ecig_nic, m4_variables_simpl,
                                                       sep="+", collapse="+"))),
                                          design=des1819_final)
summary(m4_ecig_nic1819_cox)

## Obtains main estimates
kable(round(ci.exp(m1_ecig_nic1819_cox), 2))
kable(round(ci.exp(m2_ecig_nic1819_cox), 2))
kable(round(ci.exp(m3_ecig_nic1819_cox), 2))
kable(round(ci.exp(m4_ecig_nic1819_cox), 2))

n1_ecig_nic1819_cox <- m1_ecig_nic1819_cox$model[1]
n2_ecig_nic1819_cox <- m2_ecig_nic1819_cox$model[1]
n3_ecig_nic1819_cox <- m3_ecig_nic1819_cox$model[1]
n4_ecig_nic1819_cox <- m4_ecig_nic1819_cox$model[1] #n=10181

# E-cig without nicotine status
## Runs models
m1_ecig_nonic1819_cox <- svycoxph(as.formula(paste("Surv(time, event) ~",
                          paste(ecig_nonic, collapse="+"))),
                                          design=des1819_final)
summary(m1_ecig_nonic1819_cox)
m2_ecig_nonic1819_cox <- svycoxph(as.formula(paste("Surv(time, event) ~",
                          paste(ecig_nonic, m2_variables_cox, sep="+",
                                collapse="+"))),
                                          design=des1819_final)
summary(m2_ecig_nonic1819_cox)
m3_ecig_nonic1819_cox <- svycoxph(as.formula(paste("Surv(time, event) ~",
                          paste(ecig_nonic, m3_variables_simpl, sep="+",
                                collapse="+"))),
                                          design=des1819_final)
summary(m3_ecig_nonic1819_cox)
m4_ecig_nonic1819_cox <- svycoxph(as.formula(paste("Surv(time, event) ~",
                          paste(ecig_nonic, m4_variables_simpl, sep="+",
                                collapse="+"))),
                                          design=des1819_final)
summary(m4_ecig_nonic1819_cox)

## Obtains main estimates
kable(round(ci.exp(m1_ecig_nonic1819_cox), 2))
kable(round(ci.exp(m2_ecig_nonic1819_cox), 2))
```

```
kable(round(ci.exp(m3_ecig_nonic1819_cox), 2))
kable(round(ci.exp(m4_ecig_nonic1819_cox), 2))

n1_ecig_nonic1819_cox <- m1_ecig_nonic1819_cox$model[1]
n2_ecig_nonic1819_cox <- m2_ecig_nonic1819_cox$model[1]
n3_ecig_nonic1819_cox <- m3_ecig_nonic1819_cox$model[1]
n4_ecig_nonic1819_cox <- m4_ecig_nonic1819_cox$model[1] #n=10155

# Nicotine replacement products
## Runs models
m1_nrt1819_cox <- svycoxph(as.formula(paste("Surv(time, event) ~",
                  paste(nrt, collapse="+"))),
                                     design=des1819_final)
summary(m1_nrt1819_cox)

m2_nrt1819_cox <- svycoxph(as.formula(paste("Surv(time, event) ~",
                  paste(nrt, m2_variables_cox, sep="+", collapse="+"))),
                                     design=des1819_final)
summary(m2_nrt1819_cox)

m3_nrt1819_cox <- svycoxph(as.formula(paste("Surv(time, event) ~",
                  paste(nrt, m3_variables_simpl, sep="+", collapse="+"))),
                                     design=des1819_final)
summary(m3_nrt1819_cox)

m4_nrt1819_cox <- svycoxph(as.formula(paste("Surv(time, event) ~",
                  paste(nrt, m4_variables_simpl, sep="+", collapse="+"))),
                                     design=des1819_final)
summary(m4_nrt1819_cox)

## Obtains main estimates
kable(round(ci.exp(m1_nrt1819_cox), 2))
kable(round(ci.exp(m2_nrt1819_cox), 2))
kable(round(ci.exp(m3_nrt1819_cox), 2))
kable(round(ci.exp(m4_nrt1819_cox), 2))

n1_nrt1819_cox <- m1_nrt1819_cox$model[1]
n2_nrt1819_cox <- m2_nrt1819_cox$model[1]
n3_nrt1819_cox <- m3_nrt1819_cox$model[1]
n4_nrt1819_cox <- m4_nrt1819_cox$model[1] #n=10187

## Checks proportional hazard assumption in final models

### Smoking
cox.zph.m3_smok1819_cox <- cox.zph(m3_smok1819_cox) #Note: In R version 4.1.2 shows an error: singular
cox.zph.m4_smok1819_cox <- cox.zph(m4_smok1819_cox) #Note: In R version 4.1.2 shows an error: singular

plot(cox.zph(m3_smok1819_cox), df=2)
cox.zph.m3_smok1819_cox

plot(cox.zph.m4_smok1819_cox)
cox.zph.m4_smok1819_cox
```

```r
### Snus
cox.zph.m3_snus1819_cox <- cox.zph(m3_snus1819_cox)
cox.zph.m4_snus1819_cox <- cox.zph(m4_snus1819_cox)

plot(cox.zph.m3_snus1819_cox)
cox.zph.m3_snus1819_cox

plot(cox.zph.m4_snus1819_cox)
cox.zph.m4_snus1819_cox

### E-cigarettes with nicotine
cox.zph.m3_ecig_nic1819_cox <- cox.zph(m3_ecig_nic1819_cox)
cox.zph.m4_ecig_nic1819_cox <- cox.zph(m4_ecig_nic1819_cox)

plot(cox.zph.m3_ecig_nic1819_cox)
cox.zph.m3_ecig_nic1819_cox

plot(cox.zph.m4_ecig_nic1819_cox)
cox.zph.m4_ecig_nic1819_cox

### E-cigarettes with nicotine
cox.zph.m3_ecig_nonic1819_cox <- cox.zph(m3_ecig_nonic1819_cox)
cox.zph.m4_ecig_nonic1819_cox <- cox.zph(m4_ecig_nonic1819_cox)

plot(cox.zph.m3_ecig_nonic1819_cox)
cox.zph.m3_ecig_nonic1819_cox

plot(cox.zph.m4_ecig_nonic1819_cox)
cox.zph.m4_ecig_nonic1819_cox

### Nicotine replacement therapy
cox.zph.m3_nrt1819_cox <- cox.zph(m3_nrt1819_cox)
cox.zph.m4_nrt1819_cox <- cox.zph(m4_nrt1819_cox)

plot(cox.zph.m3_nrt1819_cox)
cox.zph.m3_nrt1819_cox

plot(cox.zph.m4_nrt1819_cox)
cox.zph.m4_nrt1819_cox
```

Sensitivity analysis (iii): analyses excluding users of other forms of tobacco

We tested two approaches. The first approach (models _rw) excludes current users of snus, e-cigarettes and nicotine replacement therapy products, but keeps in the data those with missing values, as not the whole sample was asked these questions.

The second approach (models _cc) restricts the analysis to those with complete data (i.e. a much smaller sample). The comparison coefficients are with a model without excluding users but otherwise the same sample.

First approach

```r
# Smoking

## Excludes users of snus, e-cigarettes with nicotine and nicotine replacement
#products but not missing cases
finsotecc_rw_smok <- finsotecc %>%
  mutate(nicotine=1000) %>%
  mutate(nicotine = replace(nicotine, snus_status=="current user", 1)) %>%
  mutate(nicotine = replace(nicotine, ecig_nic_status=="current user", 1)) %>%
  mutate(nicotine = replace(nicotine, nrt_status=="current user", 1)) %>%
  filter(nicotine!=1)

table(finsotecc$smoking_status, finsotecc$snus_status) #looks good
table(finsotecc_rw_smok$smoking_status, finsotecc_rw_smok$snus_status) #looks good

## First column
table(finsotecc_rw_smok$smoking_status, finsotecc_rw_smok$event)

##New design object
des_rw_smok <- svydesign(id=~1, fpc=~rg_N_suomi, weights=~w_analysis_suomi,
                         strata=~rg_stratum_suomi, data=finsotecc_rw_smok)

## Creates list of variables
smok <- "factor(smoking_status)"
snus <- "factor(snus_status)"
ecig_nic <- "factor(ecig_nic_status)"
ecig_nonic <- "factor(ecig_nonic_status)"
nrt <- "factor(nrt_status)"
m2_variables <- c("factor(sex)", "pspline(age_cont)")
m3_variables <- c("factor(sex)", "pspline(age_cont)", "pspline(educ_years)",
                  "factor(maritalstatus_bin)", "factor(mother_tongue)",
                  "factor(involvement_attend_j)", "factor(fs_shp_koodi)")
m4_variables <- c("factor(sex)", "pspline(age_cont)", "pspline(educ_years)",
                  "factor(maritalstatus_bin)", "factor(mother_tongue)",
                  "factor(involvement_attend_j)", "factor(fs_shp_koodi)",
                  "pspline(bmi)")

## Run models
m1_smok_rw <- svyglm(as.formula(paste("event ~", paste(smok, collapse="+"))),
                                       design=des_rw_smok, family=quasipoisson())
summary(m1_smok_rw)
m2_smok_rw <- svyglm(as.formula(paste("event ~", paste(smok, m2_variables,
                                                        sep="+", collapse="+"))),
                                       design=des_rw_smok, family=quasipoisson())
summary(m2_smok_rw)
m3_smok_rw <- svyglm(as.formula(paste("event ~", paste(smok, m3_variables,
                                                        sep="+", collapse="+"))),
                                       design=des_rw_smok, family=quasipoisson())
summary(m3_smok_rw)
m4_smok_rw <- svyglm(as.formula(paste("event ~", paste(smok, m4_variables,
                                                        sep="+", collapse="+"))),
                                       design=des_rw_smok, family=quasipoisson())
```

```r
summary(m4_smok_rw)

#Obtains main estimates
kable(round(ci.exp(m1_smok_rw), 2))
kable(round(ci.exp(m2_smok_rw), 2))
kable(round(ci.exp(m3_smok_rw), 2))
kable(round(ci.exp(m4_smok_rw), 2))

n1_smok_rw <- m1_smok_rw$model[1]
n2_smok_rw <- m2_smok_rw$model[1]
n3_smok_rw <- m3_smok_rw$model[1]
n4_smok_rw <- m4_smok_rw$model[1] #n=41948


# Snus use
## Excludes users of tobacco, e-cigarettes with nicotine and nicotine replacement products but not miss
finsotecc_rw_snus <- finsotecc %>%
  mutate(nicotine=1000) %>%
  mutate(nicotine = replace(nicotine, smoking_status=="daily smoker", 1)) %>%
  mutate(nicotine = replace(nicotine, smoking_status=="occasional", 1)) %>%
  mutate(nicotine = replace(nicotine, ecig_nic_status=="current user", 1)) %>%
  mutate(nicotine = replace(nicotine, nrt_status=="current user", 1)) %>%
  filter(nicotine!=1)

## First column
table(finsotecc_rw_snus$snus_status, finsotecc_rw_snus$event)

##New design object
des_rw_snus <- svydesign(id=~1, fpc=~rg_N_suomi, weights=~w_analysis_suomi,
                         strata=~rg_stratum_suomi, data=finsotecc_rw_snus)

## Run models
m1_snus_rw <- svyglm(as.formula(paste("event ~", paste(snus, collapse="+"))),
                                       design=des_rw_snus, family=quasipoisson())
summary(m1_snus_rw)
m2_snus_rw <- svyglm(as.formula(paste("event ~", paste(snus, m2_variables, sep="+",
                                                        collapse="+"))),
                                       design=des_rw_snus, family=quasipoisson())
summary(m2_snus_rw)
m3_snus_rw <- svyglm(as.formula(paste("event ~", paste(snus, m3_variables, sep="+",
                                                        collapse="+"))),
                                       design=des_rw_snus, family=quasipoisson())
summary(m3_snus_rw)
m4_snus_rw <- svyglm(as.formula(paste("event ~", paste(snus, m4_variables, sep="+",
                                                        collapse="+"))),
                                       design=des_rw_snus, family=quasipoisson())
summary(m4_snus_rw)

#Obtains main estimates
kable(round(ci.exp(m1_snus_rw), 2))
kable(round(ci.exp(m2_snus_rw), 2))
kable(round(ci.exp(m3_snus_rw), 2))
kable(round(ci.exp(m4_snus_rw), 2))
```

```r
n1_snus_rw <- m1_snus_rw$model[1]
n2_snus_rw <- m2_snus_rw$model[1]
n3_snus_rw <- m3_snus_rw$model[1]
n4_snus_rw <- m4_snus_rw$model[1] #n=27113

# E-cigarettes with nicotine
## Excludes users of tobacco, snus and nicotine replacement products but not missing cases
finsotecc_rw_ecig_nic <- finsotecc %>%
  mutate(nicotine=1000) %>%
  mutate(nicotine = replace(nicotine, smoking_status=="daily smoker", 1)) %>%
  mutate(nicotine = replace(nicotine, smoking_status=="occasional", 1)) %>%
  mutate(nicotine = replace(nicotine, snus_status=="current user", 1)) %>%
  mutate(nicotine = replace(nicotine, nrt_status=="current user", 1)) %>%
  filter(nicotine!=1)

## First column
table(finsotecc_rw_ecig_nic$ecig_nic_status, finsotecc_rw_ecig_nic$event)

##New design object
des_rw_ecig_nic <- svydesign(id=~1, fpc=~rg_N_suomi, weights=~w_analysis_suomi,
                             strata=~rg_stratum_suomi, data=finsotecc_rw_ecig_nic)

## Run models
m1_ecig_nic_rw <- svyglm(as.formula(paste("event ~", paste(ecig_nic, collapse="+"))),
                                      design=des_rw_ecig_nic, family=quasipoisson())
summary(m1_ecig_nic_rw)
m2_ecig_nic_rw <- svyglm(as.formula(paste("event ~", paste(ecig_nic, m2_variables,
                                                             sep="+", collapse="+"))),
                                      design=des_rw_ecig_nic, family=quasipoisson())
summary(m2_ecig_nic_rw)
m3_ecig_nic_rw <- svyglm(as.formula(paste("event ~", paste(ecig_nic, m3_variables,
                                                             sep="+", collapse="+"))),
                                      design=des_rw_ecig_nic, family=quasipoisson())
summary(m3_ecig_nic_rw)
m4_ecig_nic_rw <- svyglm(as.formula(paste("event ~", paste(ecig_nic, m4_variables,
                                                             sep="+", collapse="+"))),
                                      design=des_rw_ecig_nic, family=quasipoisson())
summary(m4_ecig_nic_rw)

#Obtains main estimates
kable(round(ci.exp(m1_ecig_nic_rw), 2))
kable(round(ci.exp(m2_ecig_nic_rw), 2))
kable(round(ci.exp(m3_ecig_nic_rw), 2))
kable(round(ci.exp(m4_ecig_nic_rw), 2))

n1_ecig_nic_rw <- m1_ecig_nic_rw$model[1]
n2_ecig_nic_rw <- m2_ecig_nic_rw$model[1]
n3_ecig_nic_rw <- m3_ecig_nic_rw$model[1]
n4_ecig_nic_rw <- m4_ecig_nic_rw$model[1] #n=21765

# E-cigarettes without nicotine
## Excludes users of tobacco, snus and nicotine replacement products but not missing cases
finsotecc_rw_ecig_nonic <- finsotecc %>%
```

```r
  mutate(nicotine=1000) %>%
  mutate(nicotine = replace(nicotine, smoking_status=="daily smoker", 1)) %>%
  mutate(nicotine = replace(nicotine, smoking_status=="occasional", 1)) %>%
  mutate(nicotine = replace(nicotine, snus_status=="current user", 1)) %>%
  mutate(nicotine = replace(nicotine, ecig_nic_status=="current user", 1)) %>%
  mutate(nicotine = replace(nicotine, nrt_status=="current user", 1)) %>%
  filter(nicotine!=1)

## First column
table(finsotecc_rw_ecig_nonic$ecig_nonic_status, finsotecc_rw_ecig_nonic$event)

##New design object
des_rw_ecig_nonic <- svydesign(id=~1, fpc=~rg_N_suomi, weights=~w_analysis_suomi,
                               strata=~rg_stratum_suomi, data=finsotecc_rw_ecig_nonic)

## Run models
m1_ecig_nonic_rw <- svyglm(as.formula(paste("event ~", paste(ecig_nonic, collapse="+"))),
                                       design=des_rw_ecig_nonic, family=quasipoisson())
summary(m1_ecig_nonic_rw)
m2_ecig_nonic_rw <- svyglm(as.formula(paste("event ~", paste(ecig_nonic, m2_variables,
                                                              sep="+", collapse="+"))),
                                       design=des_rw_ecig_nonic, family=quasipoisson())
summary(m2_ecig_nonic_rw)
m3_ecig_nonic_rw <- svyglm(as.formula(paste("event ~", paste(ecig_nonic, m3_variables,
                                                              sep="+", collapse="+"))),
                                       design=des_rw_ecig_nonic, family=quasipoisson())
summary(m3_ecig_nonic_rw)
m4_ecig_nonic_rw <- svyglm(as.formula(paste("event ~", paste(ecig_nonic, m4_variables,
                                                              sep="+", collapse="+"))),
                                       design=des_rw_ecig_nonic, family=quasipoisson())
summary(m4_ecig_nonic_rw)

#Obtains main estimates
kable(round(ci.exp(m1_ecig_nonic_rw), 2))
kable(round(ci.exp(m2_ecig_nonic_rw), 2))
kable(round(ci.exp(m3_ecig_nonic_rw), 2))
kable(round(ci.exp(m4_ecig_nonic_rw), 2))

n1_ecig_nonic_rw <- m1_ecig_nonic_rw$model[1]
n2_ecig_nonic_rw <- m2_ecig_nonic_rw$model[1]
n3_ecig_nonic_rw <- m3_ecig_nonic_rw$model[1]
n4_ecig_nonic_rw <- m4_ecig_nonic_rw$model[1] #n=21665

# Nicotine replacement therapy products
## Excludes users of tobacco, snus and e-cigarettes but not missing cases
finsotecc_rw_nrt <- finsotecc %>%
  mutate(nicotine=1000) %>%
  mutate(nicotine = replace(nicotine, smoking_status=="daily smoker", 1)) %>%
  mutate(nicotine = replace(nicotine, smoking_status=="occasional", 1)) %>%
  mutate(nicotine = replace(nicotine, snus_status=="current user", 1)) %>%
  mutate(nicotine = replace(nicotine, ecig_nic_status=="current user", 1)) %>%
  filter(nicotine!=1)
```

```
## First column
table(finsotecc_rw_nrt$nrt_status, finsotecc_rw_nrt$event)

##New design object
des_rw_nrt <- svydesign(id=~1, fpc=~rg_N_suomi, weights=~w_analysis_suomi,
                        strata=~rg_stratum_suomi, data=finsotecc_rw_nrt)

## Run models
m1_nrt_rw <- svyglm(as.formula(paste("event ~", paste(nrt, collapse="+"))),
                                       design=des_rw_nrt, family=quasipoisson())
summary(m1_nrt_rw)
m2_nrt_rw <- svyglm(as.formula(paste("event ~", paste(nrt, m2_variables, sep="+",
                                                      collapse="+"))),
                                       design=des_rw_nrt, family=quasipoisson())
summary(m2_nrt_rw)
m3_nrt_rw <- svyglm(as.formula(paste("event ~", paste(nrt, m3_variables, sep="+",
                                                      collapse="+"))),
                                       design=des_rw_nrt, family=quasipoisson())
summary(m3_nrt_rw)
m4_nrt_rw <- svyglm(as.formula(paste("event ~", paste(nrt, m4_variables, sep="+",
                                                      collapse="+"))),
                                       design=des_rw_nrt, family=quasipoisson())
summary(m4_nrt_rw)

#Obtains main estimates
kable(round(ci.exp(m1_nrt_rw), 2))
kable(round(ci.exp(m2_nrt_rw), 2))
kable(round(ci.exp(m3_nrt_rw), 2))
kable(round(ci.exp(m4_nrt_rw), 2))

n1_nrt_rw <- m1_nrt_rw$model[1]
n2_nrt_rw <- m2_nrt_rw$model[1]
n3_nrt_rw <- m3_nrt_rw$model[1]
n4_nrt_rw <- m4_nrt_rw$model[1] #n=21943
```

Second approach

```
# Smoking

## Creates an earlier end of follow-up until December 26, 2020
finsotecc_vacc <- finsotecc %>%
  filter(

table(finsotecc$smoking_status, finsotecc$snus_status) #looks good
table(finsotecc_smok_rw2$smoking_status, finsotecc_smok_rw2$snus_status) #looks good

## First column
table(finsotecc_smok_rw2$smoking_status, finsotecc_smok_rw2$event)

##New design object
des_rw2_smok <- svydesign(id=~1, fpc=~rg_N_suomi, weights=~w_analysis_suomi,
                          strata=~rg_stratum_suomi, data=finsotecc_smok_rw2)
```

```
## Creates list of variables
smok <- "factor(smoking_status)"
snus <- "factor(snus_status)"
ecig_nic <- "factor(ecig_nic_status)"
ecig_nonic <- "factor(ecig_nonic_status)"
nrt <- "factor(nrt_status)"
m2_variables <- c("factor(sex)", "pspline(age_cont)")
m3_variables <- c("factor(sex)", "pspline(age_cont)", "pspline(educ_years)",
                  "factor(maritalstatus_bin)", "factor(mother_tongue)",
                  "factor(involvement_attend_j)", "factor(fs_shp_koodi)")
m4_variables <- c("factor(sex)", "pspline(age_cont)", "pspline(educ_years)",
                  "factor(maritalstatus_bin)", "factor(mother_tongue)",
                  "factor(involvement_attend_j)", "factor(fs_shp_koodi)",
                  "pspline(bmi)")

## Run models
m1_smok_rw2 <- svyglm(as.formula(paste("event ~", paste(smok, collapse="+"))),
                                       design=des_rw2_smok, family=quasipoisson())
summary(m1_smok_rw2)
m2_smok_rw2 <- svyglm(as.formula(paste("event ~", paste(smok, m2_variables, sep="+",
                                                         collapse="+"))),
                                       design=des_rw2_smok, family=quasipoisson())
summary(m2_smok_rw2)
m3_smok_rw2 <- svyglm(as.formula(paste("event ~", paste(smok, m3_variables, sep="+",
                                                         collapse="+"))),
                                       design=des_rw2_smok, family=quasipoisson())
summary(m3_smok_rw2)
m4_smok_rw2 <- svyglm(as.formula(paste("event ~", paste(smok, m4_variables, sep="+",
                                                         collapse="+"))),
                                       design=des_rw2_smok, family=quasipoisson())
summary(m4_smok_rw2)

#Obtains main estimates
kable(round(ci.exp(m1_smok_rw2), 2))
kable(round(ci.exp(m2_smok_rw2), 2))
kable(round(ci.exp(m3_smok_rw2), 2))
kable(round(ci.exp(m4_smok_rw2), 2))

n1_smok_rw2 <- m1_smok_rw2$model[1]
n2_smok_rw2 <- m2_smok_rw2$model[1]
n3_smok_rw2 <- m3_smok_rw2$model[1]
n4_smok_rw2 <- m4_smok_rw2$model[1] #n24666


# Snus use
## Excludes users of tobacco, e-cigarettes with nicotine and nicotine replacement products but not miss
finsotecc_rw2_snus <- finsotecc %>%
  filter(complete.cases(smoking_status, snus_status, ecig_nic_status, nrt_status)) %>%
  mutate(nicotine=1000) %>%
  mutate(nicotine = replace(nicotine, smoking_status=="daily smoker", 1)) %>%
  mutate(nicotine = replace(nicotine, smoking_status=="occasional", 1)) %>%
  mutate(nicotine = replace(nicotine, ecig_nic_status=="current user", 1)) %>%
  mutate(nicotine = replace(nicotine, nrt_status=="current user", 1)) %>%
```

```
  filter(nicotine!=1)

table(finsotecc$smoking_status, finsotecc$snus_status, useNA ="ifany" ) #looks good
table(finsotecc_rw2_snus$smoking_status, finsotecc_rw2_snus$snus_status) #looks good

## First column
table(finsotecc_rw2_snus$snus_status, finsotecc_rw2_snus$event)

##New design object
des_rw2_snus <- svydesign(id=~1, fpc=~rg_N_suomi, weights=~w_analysis_suomi,
                          strata=~rg_stratum_suomi, data=finsotecc_rw2_snus)

## Run models
m1_snus_rw2 <- svyglm(as.formula(paste("event ~", paste(snus, collapse="+"))),
                                       design=des_rw2_snus, family=quasipoisson())
summary(m1_snus_rw2)
m2_snus_rw2 <- svyglm(as.formula(paste("event ~", paste(snus, m2_variables, sep="+",
                                                        collapse="+"))),
                                       design=des_rw2_snus, family=quasipoisson())
summary(m2_snus_rw2)
m3_snus_rw2 <- svyglm(as.formula(paste("event ~", paste(snus, m3_variables, sep="+",
                                                        collapse="+"))),
                                       design=des_rw2_snus, family=quasipoisson())
summary(m3_snus_rw2)
m4_snus_rw2 <- svyglm(as.formula(paste("event ~", paste(snus, m4_variables, sep="+",
                                                        collapse="+"))),
                                       design=des_rw2_snus, family=quasipoisson())
summary(m4_snus_rw2)

#Obtains main estimates
kable(round(ci.exp(m1_snus_rw2), 2))
kable(round(ci.exp(m2_snus_rw2), 2))
kable(round(ci.exp(m3_snus_rw2), 2))
kable(round(ci.exp(m4_snus_rw2), 2))

n1_snus_rw2 <- m1_snus_rw2$model[1]
n2_snus_rw2 <- m2_snus_rw2$model[1]
n3_snus_rw2 <- m3_snus_rw2$model[1]
n4_snus_rw2 <- m4_snus_rw2$model[1] #n=21689

# E-cigarettes with nicotine
## Excludes users of tobacco, snus and nicotine replacement products but not missing cases
finsotecc_rw2_ecig_nic <- finsotecc %>%
  filter(complete.cases(smoking_status, snus_status, ecig_nic_status, nrt_status)) %>%
  mutate(nicotine=1000) %>%
  mutate(nicotine = replace(nicotine, smoking_status=="daily smoker", 1)) %>%
  mutate(nicotine = replace(nicotine, smoking_status=="occasional", 1)) %>%
  mutate(nicotine = replace(nicotine, snus_status=="current user", 1)) %>%
  mutate(nicotine = replace(nicotine, nrt_status=="current user", 1)) %>%
  filter(nicotine!=1)

## First column
table(finsotecc_rw2_ecig_nic$ecig_nic_status, finsotecc_rw2_ecig_nic$event)
```

```
##New design object
des_rw2_ecig_nic <- svydesign(id=~1, fpc=~rg_N_suomi, weights=~w_analysis_suomi,
                              strata=~rg_stratum_suomi, data=finsotecc_rw2_ecig_nic)

## Run models
m1_ecig_nic_rw2 <- svyglm(as.formula(paste("event ~", paste(ecig_nic, collapse="+"))),
                                       design=des_rw2_ecig_nic, family=quasipoisson())
summary(m1_ecig_nic_rw2)
m2_ecig_nic_rw2 <- svyglm(as.formula(paste("event ~", paste(ecig_nic, m2_variables,
                                                             sep="+", collapse="+"))),
                                       design=des_rw2_ecig_nic, family=quasipoisson())
summary(m2_ecig_nic_rw2)
m3_ecig_nic_rw2 <- svyglm(as.formula(paste("event ~", paste(ecig_nic, m3_variables,
                                                             sep="+", collapse="+"))),
                                       design=des_rw2_ecig_nic, family=quasipoisson())
summary(m3_ecig_nic_rw2)
m4_ecig_nic_rw2 <- svyglm(as.formula(paste("event ~", paste(ecig_nic, m4_variables,
                                                             sep="+", collapse="+"))),
                                       design=des_rw2_ecig_nic, family=quasipoisson())
summary(m4_ecig_nic_rw2)

#Obtains main estimates
kable(round(ci.exp(m1_ecig_nic_rw2), 2))
kable(round(ci.exp(m2_ecig_nic_rw2), 2))
kable(round(ci.exp(m3_ecig_nic_rw2), 2))
kable(round(ci.exp(m4_ecig_nic_rw2), 2))

n1_ecig_nic_rw2 <- m1_ecig_nic_rw2$model[1]
n2_ecig_nic_rw2 <- m2_ecig_nic_rw2$model[1]
n3_ecig_nic_rw2 <- m3_ecig_nic_rw2$model[1]
n4_ecig_nic_rw2 <- m4_ecig_nic_rw2$model[1] #n=21352

# E-cigarettes with nicotine
## Excludes users of tobacco, snus and nicotine replacement products but not missing cases
finsotecc_rw2_ecig_nonic <- finsotecc %>%
  filter(complete.cases(smoking_status, snus_status, ecig_nic_status, nrt_status)) %>%
  mutate(nicotine=1000) %>%
  mutate(nicotine = replace(nicotine, smoking_status=="daily smoker", 1)) %>%
  mutate(nicotine = replace(nicotine, smoking_status=="occasional", 1)) %>%
  mutate(nicotine = replace(nicotine, snus_status=="current user", 1)) %>%
  mutate(nicotine = replace(nicotine, ecig_nic_status=="current user", 1)) %>%
  mutate(nicotine = replace(nicotine, nrt_status=="current user", 1)) %>%
  filter(nicotine!=1)

## First column
table(finsotecc_rw2_ecig_nonic$ecig_nonic_status, finsotecc_rw2_ecig_nonic$event)

##New design object
des_rw2_ecig_nonic <- svydesign(id=~1, fpc=~rg_N_suomi, weights=~w_analysis_suomi,
                                strata=~rg_stratum_suomi, data=finsotecc_rw2_ecig_nonic)

## Run models
m1_ecig_nonic_rw2 <- svyglm(as.formula(paste("event ~", paste(ecig_nonic, collapse="+"))),
```

```
                                            design=des_rw2_ecig_nonic, family=quasipoisson())
summary(m1_ecig_nonic_rw2)
m2_ecig_nonic_rw2 <- svyglm(as.formula(paste("event ~", paste(ecig_nonic, m2_variables,
                                                              sep="+", collapse="+"))),
                                            design=des_rw2_ecig_nonic, family=quasipoisson())
summary(m2_ecig_nonic_rw2)
m3_ecig_nonic_rw2 <- svyglm(as.formula(paste("event ~", paste(ecig_nonic, m3_variables,
                                                              sep="+", collapse="+"))),
                                            design=des_rw2_ecig_nonic, family=quasipoisson())
summary(m3_ecig_nonic_rw2)
m4_ecig_nonic_rw2 <- svyglm(as.formula(paste("event ~", paste(ecig_nonic, m4_variables,
                                                              sep="+", collapse="+"))),
                                            design=des_rw2_ecig_nonic, family=quasipoisson())
summary(m4_ecig_nonic_rw2)

#Obtains main estimates
kable(round(ci.exp(m1_ecig_nonic_rw2), 2))
kable(round(ci.exp(m2_ecig_nonic_rw2), 2))
kable(round(ci.exp(m3_ecig_nonic_rw2), 2))
kable(round(ci.exp(m4_ecig_nonic_rw2), 2))

n1_ecig_nonic_rw2 <- m1_ecig_nonic_rw2$model[1]
n2_ecig_nonic_rw2 <- m2_ecig_nonic_rw2$model[1]
n3_ecig_nonic_rw2 <- m3_ecig_nonic_rw2$model[1]
n4_ecig_nonic_rw2 <- m4_ecig_nonic_rw2$model[1] #n=21232

# Nicotine replacement therapy products
## Excludes users of tobacco, snus and e-cigarettes but not missing cases
finsotecc_rw2_nrt <- finsotecc %>%
  filter(complete.cases(smoking_status, snus_status, ecig_nic_status, nrt_status)) %>%
  mutate(nicotine=1000) %>%
  mutate(nicotine = replace(nicotine, smoking_status=="daily smoker", 1)) %>%
  mutate(nicotine = replace(nicotine, smoking_status=="occasional", 1)) %>%
  mutate(nicotine = replace(nicotine, snus_status=="current user", 1)) %>%
  mutate(nicotine = replace(nicotine, ecig_nic_status=="current user", 1)) %>%
  filter(nicotine!=1)

## First column
table(finsotecc_rw2_nrt$nrt_status, finsotecc_rw2_nrt$event)

##New design object
des_rw2_nrt <- svydesign(id=~1, fpc=~rg_N_suomi, weights=~w_analysis_suomi,
                         strata=~rg_stratum_suomi, data=finsotecc_rw2_nrt)

## Run models
m1_nrt_rw2 <- svyglm(as.formula(paste("event ~", paste(nrt, collapse="+"))),
                                            design=des_rw2_nrt, family=quasipoisson())
summary(m1_nrt_rw2)
m2_nrt_rw2 <- svyglm(as.formula(paste("event ~", paste(nrt, m2_variables, sep="+",
                                                       collapse="+"))),
                                            design=des_rw2_nrt, family=quasipoisson())
summary(m2_nrt_rw2)
m3_nrt_rw2 <- svyglm(as.formula(paste("event ~", paste(nrt, m3_variables, sep="+",
```

```
                                                collapse="+"))),
                                        design=des_rw2_nrt, family=quasipoisson())
summary(m3_nrt_rw2)
m4_nrt_rw2 <- svyglm(as.formula(paste("event ~", paste(nrt, m4_variables, sep="+",
                                                collapse="+"))),
                                        design=des_rw2_nrt, family=quasipoisson())
summary(m4_nrt_rw2)

#Obtains main estimates
kable(round(ci.exp(m1_nrt_rw2), 2))
kable(round(ci.exp(m2_nrt_rw2), 2))
kable(round(ci.exp(m3_nrt_rw2), 2))
kable(round(ci.exp(m4_nrt_rw2), 2))

n1_nrt_rw2 <- m1_nrt_rw2$model[1]
n2_nrt_rw2 <- m2_nrt_rw2$model[1]
n3_nrt_rw2 <- m3_nrt_rw2$model[1]
n4_nrt_rw2 <- m4_nrt_rw2$model[1] #n=21557
```

Sensitivity analysis (iv): analyses exploring heterogeneity by start of vaccination

COVID-19 vaccination could affect the risk of COVID-19 incidence. We unfortunately do not currently have individual data on vaccination status. We carried out post-hoc sensitivity analyses include an exploratory assessment of whether our results could differ by the periods before and after the emergence of the COVID-19 vaccination. We consider the formal start of the vaccination on December 26, 2020, although rollout only expanded significantly in the coming months.

```
# Considers cases after Dec 31 as uncensored
finsotecc_prevac <- finsotecc %>%
   mutate(event = replace(event, end_date>="2020-12-26", 0))

# Considers cases before Dec 31 as uncensored
finsotecc_postvac <- finsotecc %>%
   mutate(event = replace(event, end_date<"2020-12-26", 0))

#Checks
table(finsotecc$end_date, finsotecc$event)
table(finsotecc_postvac$end_date, finsotecc_postvac$event)
table(finsotecc$smoking_status, finsotecc$event)
table(finsotecc_postvac$smoking_status, finsotecc_postvac$event)

# Re-runs the main analysis with the new definition
## Smoking status

## Create a subset where participants with missing data on smoking
#(smoking_status==NA) are excluded
finsotecc_postvac <- within(finsotecc_postvac, smoking_status <- relevel(smoking_status,
                                                    ref = 'never smoker'))
des_final_postvac <- svydesign(id=~1, fpc=~rg_N_suomi, weights=~w_analysis_suomi,
                         strata=~rg_stratum_suomi, data=finsotecc_postvac)

finsotecc_prevac <- within(finsotecc_prevac, smoking_status <- relevel(smoking_status,
```

```
                                                         ref = 'never smoker'))
des_final_prevac <- svydesign(id=~1, fpc=~rg_N_suomi, weights=~w_analysis_suomi,
                       strata=~rg_stratum_suomi, data=finsotecc_prevac)

## Table S10 Smoking first column
table(finsotecc_prevac$smoking_status, useNA = "ifany")
table(finsotecc_postvac$smoking_status, useNA = "ifany")
table(finsotecc_prevac$smoking_status, finsotecc_prevac$event, useNA = "ifany")
table(finsotecc_postvac$smoking_status, finsotecc_postvac$event, useNA = "ifany")

## Crude model
m1_prevac <- svyglm(event ~ factor(smoking_status), design=des_final_prevac,
                    family=quasipoisson())
summary(m1_prevac)

m1_postvac <- svyglm(event ~ factor(smoking_status), design=des_final_postvac,
                     family=quasipoisson())
summary(m1_postvac)

## Adjusted for age and sex
m2_prevac <- svyglm(event ~ factor(smoking_status) + factor(sex) + pspline(age_cont),
                       design=des_final_prevac, family=quasipoisson())
summary(m2_prevac)

m2_postvac <- svyglm(event ~ factor(smoking_status) + factor(sex) + pspline(age_cont),
                       design=des_final_postvac, family=quasipoisson())
summary(m2_postvac)

## Adjusted for confounders
m3_prevac <- svyglm(event ~ factor(smoking_status) + factor(sex) + pspline(age_cont) +
                       pspline(educ_years) + factor(maritalstatus_bin) +
                       factor(mother_tongue) + factor(involvement_attend_j) +
                       factor(fs_shp_koodi),
                       design=des_final_prevac, family=quasipoisson())
summary(m3_prevac)

m3_postvac <- svyglm(event ~ factor(smoking_status) + factor(sex) + pspline(age_cont) +
                       pspline(educ_years) + factor(maritalstatus_bin) +
                       factor(mother_tongue) + factor(involvement_attend_j) +
                       factor(fs_shp_koodi),
                       design=des_final_postvac, family=quasipoisson())
summary(m3_postvac)

#Obtains main estimates
kable(round(ci.exp(m2_prevac), 2))
kable(round(ci.exp(m2_postvac), 2))
kable(round(ci.exp(m3_prevac), 2))
kable(round(ci.exp(m3_postvac), 2))

n2_smok_prevac <- m2_prevac$model[1]
n3_smok_prevac <- m3_prevac$model[1] #looks good, n=44199
n2_smok <- m2_postvac$model[1]
n3_smok <- m3_postvac$model[1] #looks good, n=44199
```

```r
# Snus

## Table S10: First column
table(finsotecc_prevac$snus_status, useNA = "ifany")
table(finsotecc_prevac$snus_status, finsotecc_prevac$event, useNA = "ifany")
table(finsotecc_postvac$snus_status, useNA = "ifany")
table(finsotecc_postvac$snus_status, finsotecc_postvac$event, useNA = "ifany")


## Crude model
m1_prevac_snus <- svyglm(event ~ factor(snus_status),
                         design=des_final_prevac, family=quasipoisson())
summary(m1_vacc_snus)

m1_postvac_snus <- svyglm(event ~ factor(snus_status),
                         design=des_final_postvac, family=quasipoisson())
summary(m1_postvac_snus)



## Adjusted for age and sex
m2_prevac_snus <- svyglm(event ~ factor(snus_status) + factor(sex) + pspline(age_cont),
                         design=des_final_prevac, family=quasipoisson())
summary(m2_prevac_snus)

m2_postvac_snus <- svyglm(event ~ factor(snus_status) + factor(sex) + pspline(age_cont),
                         design=des_final_postvac, family=quasipoisson())
summary(m2_postvac_snus)

## Adjusted for confounders
m3_prevac_snus <- svyglm(event ~ factor(snus_status) + factor(sex) + pspline(age_cont) +
                      pspline(educ_years) + factor(maritalstatus_bin) +
                      factor(mother_tongue) + factor(involvement_attend_j) +
                      factor(fs_shp_koodi),
                      design=des_final_prevac, family=quasipoisson())
summary(m3_prevac_snus)

m3_postvac_snus <- svyglm(event ~ factor(snus_status) + factor(sex) + pspline(age_cont) +
                      pspline(educ_years) + factor(maritalstatus_bin) +
                      factor(mother_tongue) + factor(involvement_attend_j) +
                      factor(fs_shp_koodi),
                      design=des_final_postvac, family=quasipoisson())
summary(m3_postvac_snus)

#Obtains main estimates
kable(round(ci.exp(m2_prevac_snus), 2))
kable(round(ci.exp(m2_postvac_snus), 2))
kable(round(ci.exp(m3_prevac_snus), 2))
kable(round(ci.exp(m3_postvac_snus), 2))

# Checking that estimates are on the same sample
n1_snus_prevac <- m1_prevac_snus$model[1]
```

```
n2_snus_prevac <- m2_prevac_snus$model[1]
n3_snus_prevac <- m3_prevac_snus$model[1] #looks good, n=32931
n1_snus <- m1_postvac_snus$model[1]
n2_snus <- m2_postvac_snus$model[1]
n3_snus <- m3_postvac_snus$model[1] #looks good, n=32931
```

End of script

Please contact me if you find errors or have any feedback on the code at @spenafajuri (Twitter) or sebastian.penafajuri@thl.fi
